# Evaluating the Quality of Colorectal Cancer Care across the Interface of Healthcare Sectors

**DOI:** 10.1371/journal.pone.0060947

**Published:** 2013-05-01

**Authors:** Sabine Ludt, Elisabeth Urban, Jörg Eckardt, Stefanie Wache, Björn Broge, Petra Kaufmann-Kolle, Günther Heller, Antje Miksch, Katharina Glassen, Katja Hermann, Regine Bölter, Dominik Ose, Stephen M. Campbell, Michel Wensing, Joachim Szecsenyi

**Affiliations:** 1 Department of General Practice and Health Services Research, University of Heidelberg Hospital, Heidelberg, Germany; 2 AQUA-Institute for Applied Quality Improvement and Research in Healthcare, Goettingen, Germany; 3 Institute for Population Health – Centre for Primary Care, University of Manchester, Manchester, United Kingdom; 4 Scientific Institute for Quality of Healthcare, Radboud University Nijmegen Medical Centre, Nijmegen, The Netherlands; Davidoff Center, Israel

## Abstract

**Background:**

Colorectal cancer (CRC) has a high prevalence in western countries. Diagnosis and treatment of CRC is complex and requires multidisciplinary collaboration across the interface of health care sectors. In Germany, a new nationwide established program aims to provide quality information of healthcare delivery across different sectors. Within this context, this study describes the development of a set of quality indicators charting the whole pathway of CRC-care including data specifications that are necessary to operationalize these indicators before practice testing.

**Methods:**

Indicators were developed following a systematic 10 step modified ‘RAND/UCLA Appropriateness Method’ which involved a multidisciplinary panel of thirteen participants. For each indicator in the final set, data specifications relating to sources of quality information, data collection procedures, analysis and feedback were described.

**Results:**

The final indicator set included 52 indicators covering diagnostic procedures (11 indicators), therapeutic management (28 indicators) and follow-up (6 indicators). In addition, 7 indicators represented patient perspectives. Primary surgical tumor resection and pre-operative radiation (rectum carcinoma only) were perceived as most useful tracer procedures initiating quality data collection. To assess the quality of CRC care across sectors, various data sources were identified: medical records, administrative inpatient and outpatient data, sickness-funds billing code systems and patient survey.

**Conclusion:**

In Germany, a set of 52 quality indicators, covering necessary aspects across the interfaces and pathways relevant to CRC-care has been developed. Combining different sectors and sources of health care in quality assessment is an innovative and challenging approach but reflects better the reality of the patient pathway and experience of CRC-care.

## Introduction

Colorectal cancer (CRC) is the most common cancer and the second leading cause of cancer related death in Europe [1]. Besides lung and breast cancer it is the third most common cancer worldwide [2]. Annually, there are approximately 70.000 incidences and 30.000 cases of death of both, men and women, related to CRC in Germany [3].

The pathway of care for patients with CRC is complex involving multiple interfaces and multidisciplinary health care providers in inpatient and outpatient settings, relating to diagnostic procedures, therapy decision-making, multimodal treatment and surveillance. Beside the expertise of health care providers, coordination and communication and a good infrastructure for surveillance and follow-up are necessary to provide good quality throughout the entire pathway of care.

Transitions between hospital and ambulatory care are the most vulnerable parts of the delivery of high quality and safe care, especially in fragmented health care structures such as are established in Germany and in the United States [4], [5].

Quality of colorectal cancer care is an important clinical and political issue worldwide [5], [6]. As quality-measurement of processes and outcomes has an important role in many strategies to improve healthcare, much effort has gone into developing and applying quality indicators over the last decades [7]. Quality indicators are defined as measurable elements of practice performance for there is evidence or consensus that they can be used to assess and change the quality of care provided [7]. It is important that quality indicators meet a range of requirements such as relevance, validity, reliability and feasibility relating to the implementation of indicators in routine care [8].

Whereas the development of quality indicators for colorectal cancer care has been reported in many countries [9]–[13], operationalization of these indicators including specification of data sources, data collection methods and analyzing or practice testing have been rarely described [14]. Previously developed quality indicators and quality improvement initiatives have been focused mainly on surgical treatment reflecting the importance of primary tumor resection as a curative approach within multimodal therapy regimens [6]. However, quality assessment from a comprehensive disease perspective, measuring the quality of CRC care from patient presentation to postoperative surveillance and follow-up throughout the entire pathway of CRC-care, is not yet described [5], [13], [15].

Patient centered care is as an integral part of evaluating health care [16] particularly in cancer care [5], [17]. Previous literature shows that professionals' opinions about high quality care may deviate from patients' perspectives, so it is necessary to involve patients in indicator development [18]. However, many sets of quality indicators do not include measures of patient centeredness or experience.

A wide variety of methodological approaches for developing quality indicators has been reported; however, patient representatives are mostly not included and practice testing of indicators is not always provided during the development process [19]. It is also crucial to test sets of indicators using a testing protocol [20], [21].

Therefore, the aim of this study was twofold: First, to develop a comprehensive set of cross-sectoral quality indicators along the whole pathway of colorectal cancer care including patient representatives; Second, to describe important steps towards practice testing of these indicators such as specification of data management, analyzing and feedback procedures.

## Methods

### Setting

Germany has a population of about 82 million inhabitants, about 90 percent of the population is covered by statutory insurance (generally under compulsory insurance cover), while private insurance - to which only civil servants, the self-employed and high-earning employees have access - covers about 10 percent of the population. The costs of statutory health insurance are split roughly 50:50 between employers and employees, with the government paying for coverage of welfare recipients [22].

Health spending accounted for 11.6% of the gross domestic product (GDP) in Germany in 2010, more than two percentage points higher than the Organization for Economic Co-operation and Development (OECD) average of 9.5%. Still, health spending as a share of GDP remains much lower in Germany than in the United States (which spent 17.6% of its GDP on health in 2010) [23].

The German Health Care system is characterized by the fragmentation of care structures with rigid financial barriers between ambulatory and hospital care. The almost 250 health insurance funds and their umbrella organizations regulate the system. In the ambulatory sector fund members have the right of a free choice of doctor and can consult a specialist directly [22].

The Federal Joint Committee (G-BA) regulates the healthcare system independently under the supervision of the Ministry of Health. In 2009, the G-BA established a comprehensive program for quality improvement across healthcare sectors in Germany (‘Sektorenübergreifende Qualitätssicherung im Gesundheitswesen’ or ‘SQG’) and commissioned an independent institution, the Institute for Applied Quality Improvement and Research in Health Care (AQUA-Institute) [24] to develop national cross-sectoral quality measures, data collection procedures and analytic procedures to feed-back measurement results to health care providers to stimulate quality improvement [25]. As the SQG quality program is obligatory and is nationwide, health care providers in both sectors are required to record and transfer quality information.

### Development process

The study was carried out between January 2010 and December 2011. The AQUA-institute processed this task in collaboration with the ‘Department of General Practice and Health Services Research’ at the University Hospital at Heidelberg. A ten step [25] modified ‘RAND/UCLA Appropriateness Method’ [26] was applied to develop the quality measures. This procedure included a scoping workshop with experts, structured literature search to identify quality indicators, two rounds of panel-ratings, design of measure specifications and the delivery of a final report to be approved by the G-BA (Table 1).

**Table 1:** Phases and steps in the development and testing of indicators for colorectal cancer care.

Phases	Steps
Planning	1. Scoping workshop
	- Collecting existing knowledge and practice
	2. Structured search
	- Structured literature search using a predefined search model
	- Structured search in indicator agencies
	3. Organization of the assessment panel
	- 11 multidisciplinary experts and 2patient representatives
	4. Preparation of quality indicators for the panel assessment
	- Defining of the indicator (numerator, denominator)
	- Inclusion and exclusion criteria
	- Target levels or standards
	- Type (process, outcome, intermediate outcome, structure)
	- Sources
	- Evidence
Rating	5. Preliminary meting
	- Overview about the development procedures
	- Providing of indicator templates
	6. Rating rounds
	- Validity – postal and meeting
	- Feasibility – postal and meeting
Operationalizing	7. Specification of measures
	- Unit of analysis (patient, hospital, provider)
	- Data sources (administrative data, medical record data, survey)
	- Risk adjustment
	- Responsibility for indicator results
	- Data sources
	- Data collection procedures
	- Analytical plan
	- Feedback strategies
Approval	8. Approval of the final report by the G-BA
Piloting	9. Feasibility test
	10. Field testing

### Scoping workshop

Members of medical societies and interest groups involved in the CRC-care process were openly invited to a scoping workshop by post and via a website announcement. 55 experts of various clinical professions such as surgery, gastroenterology, oncology, pathology, family medicine, human genetics, epidemiology, nursing and patient representatives participated in the meeting. Representatives of the federal association of sickness funds and other stakeholders of the German healthcare system reported on quality improvement initiatives. The aim of the workshop was to collect and synthesize knowledge of experts across the CRC healthcare interfaces.

### Structured search for indicators

The search consisted of 3 steps: 1) a preliminary search to get an overview about current colorectal cancer care and the situation in Germany (Table S2), 2) the main systematic literature search to identify internationally applied quality indicators (Tables S3, S4, S5, S6) and 3) a search of international agencies and indicator databases to identify quality indicators concerning colorectal cancer care (Table S7).

In a preliminary search, Cochrane Database of Systematic Reviews, guideline databases, Medline and oncology journals were searched for guidelines and systematic reviews on CRC and a final search model for a systematic search in Medline was developed. We identified 28 papers from the ‘Cochrane Colorectal Cancer group’ and 45 international guidelines on CRC including one German evidence-based guideline [3], 1 Health Technology Assessment (HTA)- report and 25 additional papers that highlighted the German perspective.

**Figure 1:**
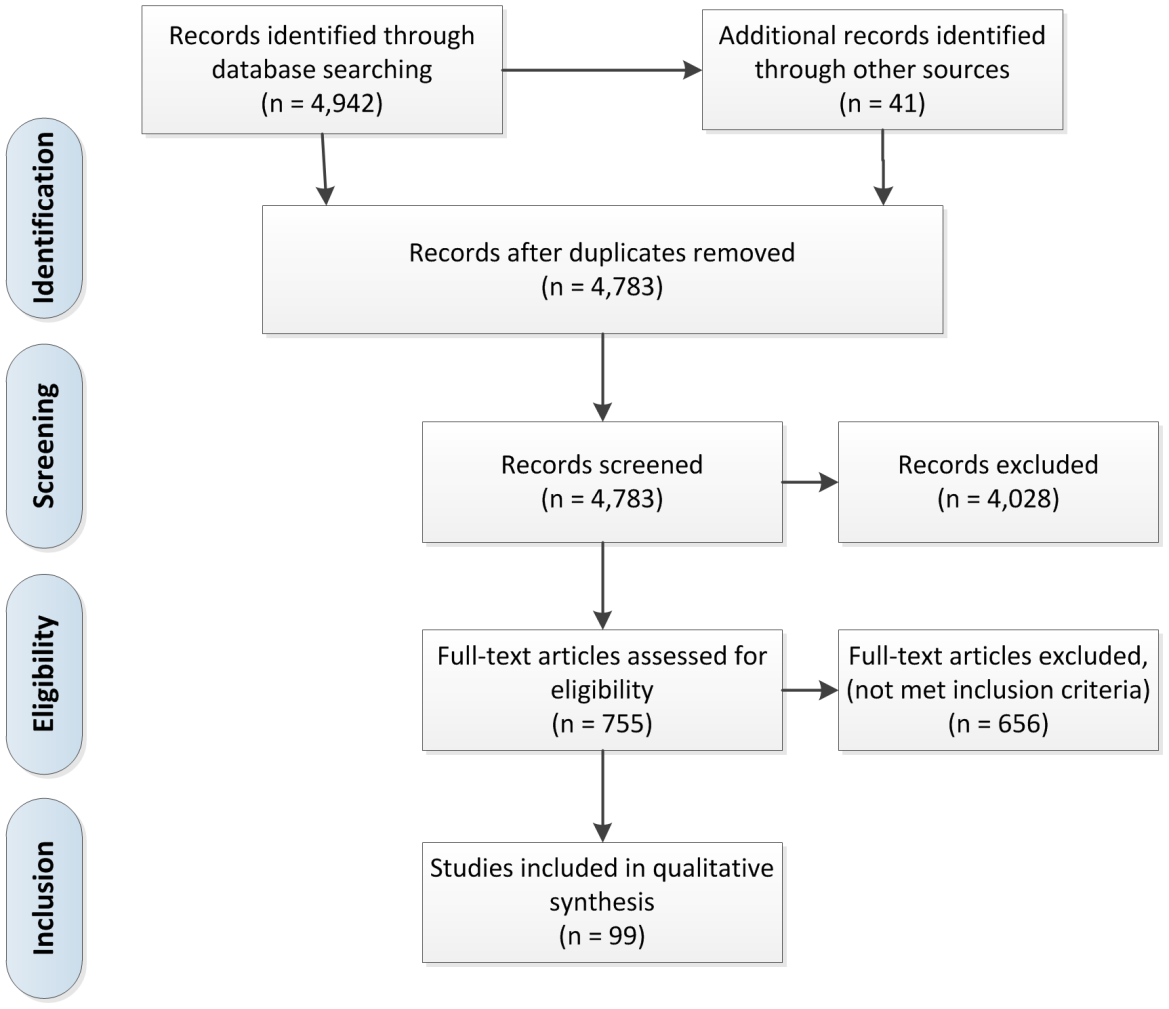
Systematic literature search - Flow chart.

The structured search was carried out from February to March 2010. We searched MEDLINE® (from 1998 to March 2010) systematically using a predefined search strategy (Tables S3) and identified 4,942 potentially relevant abstracts (Table S4). Additionally, 41 relevant publications were found by hand search of peer reviewed oncology journals. Paired reviewers (researchers including physicians and methodologists) screened the abstracts independently using predefined inclusion and exclusion criteria (Table S5) and ordered the full-text when either reviewer selected it for inclusion. The full-texts were abstracted for quality indicators using self-developed and piloted abstraction forms. Finally, 99 publications (Table S6) met inclusion criteria, of which 289 quality indicators for the diagnosis and treatment of colorectal cancer were extracted (Figure 1).

**Figure 2:**
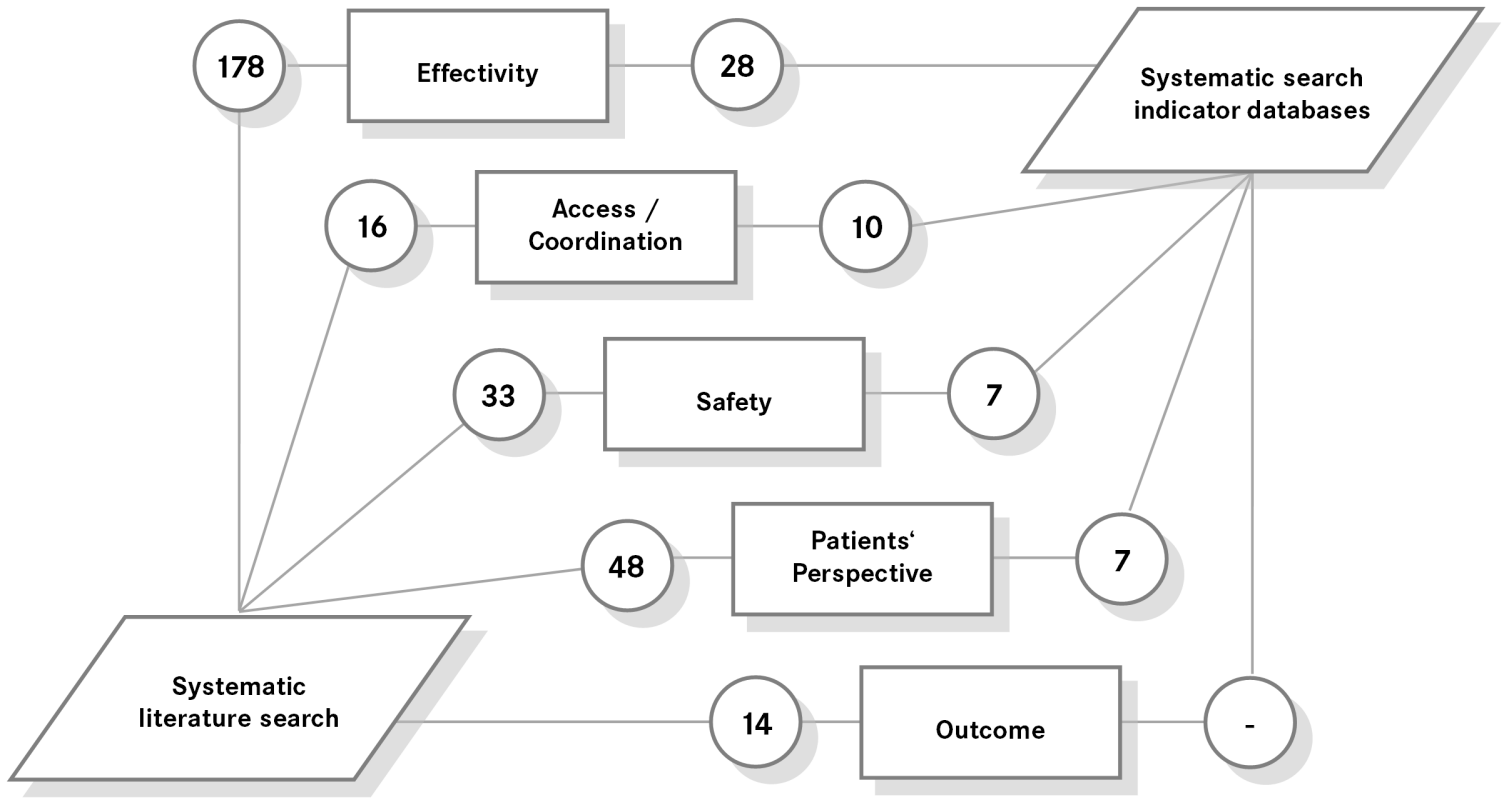
N^o^ of quality indicators identified by systematic search (allocated to the OECD quality model dimensions) [**24**].

A structured search of indicator agencies worldwide (73 previously identified agencies were retrieved for indicators) identified 419 quality indicators (Table S7) in various dimensions [27]. Indicators were also extracted from national grey literature, such as professional-society documents (German Cancer Society) or government reports. After removing duplicates, 52 quality indicators remained (Figure 2).

### Preparing candidate indicators for expert panel ratings

The resulting 341 quality indicators composed of 289 indicators from systematic literature search and 52 indicators from indicator agencies, were translated into German and allocated to the clinical dimensions ‘diagnosis’, ‘therapy’, ’management/coordination of care’, ‘patient perspective’ and ‘outcome’ as appropriate. The indicators were drafted to 210 self-developed standardized templates providing original indicator wordings (English mostly) and German translations. Indicators that differed from each other only slightly in wording were subsumed to one single indicator template providing the various original wordings. Additionally, templates included categories for a short description of the indicator, the definition of numerator and denominator, inclusion and exclusion criteria, indicator level targets and the type of the quality indicator relating to structure, process or outcome. Sources of the indicators and evidence from literature or guidelines were also provided. Finally, 210 templates summarized 341 quality indicators in the clinical dimensions diagnosis (22), therapy (104), management and coordination of care (47), patient perspective (31) and outcome (6).

### Expert panel ratings

The expert panel ratings were carried out between June and September 2010. All medical societies involved in diagnosis and treatment of CRC were asked to inform their members to apply for the panel. Furthermore, an invitation was announced at the scoping workshop in Heidelberg and also provided openly via internet [24]. From 77 applications for the panel, 14 experts were selected according to predefined criteria aligned to including the most relevant disciplines in the pathway of CRC care. If experts were equally qualified, the panel candidate was drawn randomly by lot. Finally, these multidisciplinary experts from inpatient and outpatient care were chosen for the panel: One family physician, one gastroenterologist, two clinical oncologists, one psychotherapist/psycho-oncologist, three visceral surgeons, one pathologist, one representative of a regional consortium/working group for quality assurance, one expert for quality assurance in oncology, and one representative of a regional cancer registry. Additionally, two patient representatives nominated by the G-BA completed the panel membership. As no radiation oncologist applied to the panel, a radiation oncologist was nominated by the German Society of Radiation Oncology (DEGRO) to give advice to the panel. All panel members had to declare conflicts of interests in a written form.

The panel rating was performed in two rounds consisting of a postal rating and a face-to-face panel meeting in each round. The voting of all members of the panel was counted equally.

In round one, panelists rated the content validity in terms of *relevance* on a 9-point integer scale with a score of one (not at all relevant) for the first answer up to nine (very relevant). In the postal ratings, the quality indicators were rated by each expert of the panel at home/office and sent back anonymously by return envelope. The indicator templates provided the opportunity to give comments to adapt indicators if necessary. At the two-day panel meeting, the results of the postal ratings from round one were presented and discussed. If necessary, the quality indicators were modified to align them to recommendations of the German evidence based guideline [3] or to the German health care system. After discussion, each quality indicator was re-rated.

In round two, the same procedures were applied to rate the *feasibility* of the indicators.

Analyses of the ratings were based on the ‘RAND/UCLA’ Appropriateness Method [26]. For each quality indicator overall panel median scores and the level of agreements within the panel were calculated. Median scores of 7–9 and consensus of more than 75% were defined as “agreement”, the quality indicator was classified as valid. A median score of 1–3 with an agreement of more than 75% was defined as not valid. In the feasibility rating, a quality indicator with a median score of more than 4 was defined as feasible. All statistical analyses were performed with SPSS-Statistics/PASW (Predictive Analysis Soft Ware) Vs 18.

### Designing of measure specifications

For each indicator, data sources and required data fields were specified including trigger criteria to identify patients for the quality assurance program, data fields to create the indicator and data fields that were required for risk adjustment where appropriate. Trigger criteria were derived from the International classification of Diseases (ICD-10 GM) – codes that were available in the ambulatory and in the hospital sector, and from process codes of the hospital (OPS codes) and ambulatory (fee schedule items) reimbursement systems. Furthermore, data fields were described that had to be additionally recorded for quality- assurance purposes. Specifications for data flow and analyses were provided. To create indicators requiring data from both the ambulatory and the hospital sector, it had to be ensured that all data would come together in a separate trusted center that had to assign this information to a certain patient and to forward these data anonymously to the AQUA-institute for analysis. To collect data relating to the indicators on patients' perspectives, procedures for random selection of patients and implementation of patient surveys during the therapy course were described. Peer review auditing as an innovative concept of data collection methods was described for indicators reflecting the quality of reports that summarize important clinical findings such as the colonoscopy-report or the surgery-report. Data specifications and data flow models were discussed with the G-BA and revised before being included in a final report.

## Results

### Final set of quality indicators

The final set included 52 quality indicators (Table 2 and Table S1) representing significant in- and outpatient procedures along the entire pathway of CRC care. The set of indicators described pre-therapeutic diagnostic procedures (11 indicators), therapeutic procedures (28 indicators), surveillance (2 indicators) and outcomes (4 indicators). Furthermore, 7 indicators were related to patient specific issues (Table 2).

**Table 2:** Quality indicators of CRC care included in the final set (for detailed description see Table S1).

Clinical Dimension	Indicator		Data sources*	Feed-back**
Diagnostic procedures and staging	1	Availability and constitution of multidisciplinary tumor boards/ambulatory multidisciplinary teams	1; 2;3	11
	2	Pre-therapeutic assessment of CRC-patients by tumor boards/ambulatory multidisciplinary teams	5 or 6	11
	3	Tumor boards/ambulatory multidisciplinary teams with expertise in metastatic surgery	1;2; 3	11
	4	Availability and content of a preoperative colonoscopy report	4	10
	5	Colonoscopy reports with documentation of specific quality aspects	7	10
	6	Pre-therapeutic availability of a histo-pathologic diagnosis (tumor biopsy)	1;4 or 6	10
	7	Pre-therapeutic liver imaging in CRC patients	1;4 or 6	10
	8	Pre-therapeutic rigid rectoscopy in RC-patients	1;4 or 6	10
	9	Pre-therapeutic staging using cTNM-categories in RC-patients	1;4 or 6	10
	10	Pre-therapeutic pelvis imaging using multi-slice CT or high-resolution MRI in RC-patients	1;4 or 6	10
	11	Pre-therapeutic imaging of liver and lungs using CT or MRI in CRC-patients with liver metastases	1;4 or 6	10
Pre-operative manage-ment	12	Pre-operative assessment of bowel; urinary and sexual function in RC-patients	1; 8	10
	13	Assessment of Bethesda-criteria in CRC-patients	1; 5	
	14	Pre-operative stoma education where appropriate	1; 5	
	15	Preoperative marking of stoma localization	1; 5	
Radio- (chemo)therapy	16	Neo-adjuvant radio(chemo)therapy in RC-patients	1; 5	11
	17	Radiotherapy according to quality standards of the German Society of Radiation Oncology (DEGRO) in RC-patients	1;2;3	10
Surgery and histo-pathologic exami-nation	18	Antibiotic prophylaxis before CRC-surgery	1;5 or 7	10
	19	En bloc resection in case of tumor adherence to other organs	1;3 or 7	10
	20	Intraoperative exploration of liver and peritoneal lining	1;3, or 7	10
	21	Intraoperative local dissemination of tumor cells	1;3 or 7	10
	22	Total/partial mesorectal excision (TME/PME) in RC-patients	1;3 or 7 or 6	10
	23	Abdominal perineal resection (APR) in RC-patients	1;3 or 7 or 6	10
	24	Major anastomotic leakage after elective CRC-surgery	1; 3 or 7	10
	25	Surgical re- interventions after CRC-surgery	1;3 or 7	10
	26	Examination of least 12 lymph nodes	1;2; 3	10
	27	Rate of local R0-resections in CRC-patients	1;2; 3	10
	28	Rate pT1 carcinomas in CRC-patients	1;2; 3	11
	29	Liver- and lung-metastasectomy in patients with stage IV CRC	1;2; 3	
	30	Documentation of distal tumor-free margin in RC-patients	1;2; 3	10
	31	Mesorectal CRM-positive (CRM <1mm) radical surgical resection in RC-patients	1;2; 3	
	32	Quality of Total Mesorectal Excision (TME)	1;2; 3	
	33	Pathology reports following quality standards of the German Society of Pathology	1;2; 3 or 7	
	41	Examination of microsatellite-instability in CRC-patients younger than 50 years	1;2; 3	
Post-operative manage-ment	35	Post-operative assessment of bowel; urinary and sexual function in RC-patients	8	10
	36	Providing of information and instructions about stoma management in patients with stoma	8	
Adjuvant chemo-therapy	37	Adjuvant chemotherapy in patients with stage III CC	1;2; 5	11
	38	Time interval between surgery and starting adjuvant chemotherapy in patients with stage III CC	1;2; 5	
	39	Documentation of chemotherapy treatment summary in medical records and passing on this information to the patient and to the physician providing surveillance	1;2; 3	10
Sur-veillance	42	Postoperative colonoscopy within 6 months in patients with incomplete preoperative colonoscopy	1;2; 5 or 6	11
	43	Postoperative surveillance as recommended in the German S3 guideline	1; 2; 4 or 6	
Patient perspec-tive	40	Delivery of a written plan for pain management in CRC-patients where appropriate	8	10
	44	Sharing the decision with the patient regarding therapeutic procedures	8	10
	45	Opportunities to ask the specialists questions	8	10
	46	The patient is offered contact with a companion in distress	8	10
	47	The patient knows, which activities are allowed at home	8	10
	48	The patient knows, which side effects or complications to be aware of at home	8	10
	49	The patient knows, when to contact the general practitioner or specialist	8	10
Outcomes	50	5-year overall survival in CRC-patients	9	11
	51	5-year local recurrence RC-patients	1; 2; 3 or 6	
	52	30-day-mortality after primary CRC-surgery	9	10
	53	Assessment of quality of life with a specific instrument in CRC-patients	8	11

* Data sources: 1: Inpatient administrative and/or reimbursement data (OPS-codes), 2: Outpatient administrative and/or reimbursement data (fee schedule items), 3: Prospectively collected clinical data, 4: Retrospectively collected clinical data during tracer procedure, 5: Medical record, 6: Implementation of new procedure codes: OPS-codes (hospital) or fee schedule items (ambulatory sector), 7: Peer review, 8: Patient survey, 9: Administrative data (sickness funds).

** Feedback: 10: Healthcare provider level – Benchmarking feedback reports with ‘structured dialogue’ in case of poor results, 11: Area level – multidisciplinary discussion.

### Diagnostic procedures

In line with the German evidence-based guideline [3], all relevant diagnostic procedures such as colonoscopy, biopsy or imaging techniques were covered by the indicators. Most of these indicators were process indicators, measuring whether a diagnostic procedure has been performed such as a pre-therapeutic tumor biopsy (indicator 7). Additionally, the diagnostic indicator set included technical measures pertaining to specific details of a procedure such as the availability of standardized colonoscopy reports indicating not only performance but also the quality of colonoscopy-performance and report (indicator 5). The availability of multidisciplinary tumor boards for therapy decision-making, as an example for a cross-sectoral indicator, was also included (indicator 1 and 2).

### Therapeutic indicators

Therapeutic procedures comprised surgery procedures, pre- and post-therapeutic management and the application of radiotherapy and chemotherapy. Most indicators concerned surgical processes, reflecting the importance of colorectal resection for cancer as a curative approach. Beside process measures, technical measures such as the delivery and quality of a total mesorectal excision (TME) in patients with RC were also included (indicators 22 and 32). The assessment of the pre- and postoperative functional bowel status (indicators 12 and 35) reflected patient relevant issues. The quality of staging procedures was described by two indicators including the quality of the pathology report according to the standards of the ‘German Pathology Society’. Radiotherapy was represented by two indicators (indicators 16 and 17), with one describing a technical measure providing information about the quality of radiotherapy performance (indicator 17). Three general indicators were related to chemotherapy (indicators 37, 38, 39). No technical measure for chemotherapy was identified.

Follow-up indicators were related to surveillance colonoscopy (indicator 42) and other diagnostic procedures recommended for the early detection of disease recurrence (indicator 43). Outcome indicators were related to mortality rates (indicator 50 and 52), disease recurrence for RC (indicator 51) and the quality of life as a patient related outcome indicator (indicator 53).

Across these processes, 7 indicators representing patients' perspectives completed the set: These indicators were related to patient information, shared decision-making, support, pain management and follow-up management.

### Excluded indicators

Indicators were excluded during the panel rounds for several reasons: Some indicators were rated not valid as they were deemed to be not specific enough for CRC, such as indicators addressing colorectal surgery in general. Other indicators were seen as duplicates of other included indicators and therefore redundant. Furthermore, indicators were excluded if their measurement was assumed to be very resource intensive such as the proportion of RC-patients in appropriate UICC-stages receiving adjuvant chemotherapy without having received neo-adjuvant radio (chemo) therapy before cancer resection. Indicators reflecting patients' perspectives were suggested to be difficult to assess and to feed-back to providers as indicator results were not unambiguously attributable to a specific healthcare provider.

Indicator 34, concerning pain management, was excluded by AQUA after the panel rounds, as this issue was already addressed in a generic part of the patient survey within all SQG programs [24].

### Operationalization of indicators

To identify eligible patients for inclusion in the CRC quality assurance program, two tracer events were defined: 1) primary tumor resection delivered in hospital and 2) neo-adjuvant chemotherapy that can be provided either ambulatory or in hospital. These tracer procedures were used to index eligible patients for follow up. Diagnostic procedures and findings prior to these tracer events had to be recorded retrospectively in the medical charts.

We identified four sources of quality information to be used in combination to create the quality indicators: first, patient charts - requiring additional documentation on clinical patient information, such as co-morbidities; second, administrative and reimbursement inpatient data (ICD codes and OPS-codes) and outpatient data (ICD codes and fee schedule items) to collect information on diagnoses and procedures; third; administrative data from sickness-funds, mainly to collect information on vital status and fourth, patient survey data to assess patients' perspectives. Data abstraction protocols were developed. Furthermore, a method to arrange patient surveys including self-developed questionnaires and validated instruments for the assessment of quality of life and functional bowel status were developed.

### Feedback procedures

Two groups of feedback procedures were identified. First, indicator results that could be ascribed unambiguously to healthcare providers or facilities were targeted to be embedded in established feedback procedures within the German SQG-program [24]. These procedures included the provision of a benchmarking quality report and a ‘structured dialogue’ with healthcare providers achieving poor results to identify quality problems.

Providing feedback for the second group of indicators, which reflected cross-sectoral multidisciplinary coordination and shared responsibilities such as the time period up to starting chemotherapy after surgical resection, was more complex. For this group of indicators (area indicators) it was proposed to address feedback, not to single healthcare providers or facilities but, to define reference regions such as diversion areas of hospitals and to provide feedback within multidisciplinary quality circles to promote quality improvement.

### Final report

The final report comprised the detailed description of the methodology, the final set of quality indicators and data abstraction forms for each indicator according to established data sources and structures within the German healthcare system. Furthermore, alternative implementation methods were proposed and discussed in order to reduce data collection time and effort, such as peer review auditing and the implementation of specific reimbursement codes (OPS-codes or fee schedule items). The final report was approved by the G-BA in December 2011.

## Discussion

During this study, a set of 52 quality indicators was developed to reflect the entire pathway of colorectal cancer care. Data specifications for the final set of indicators were developed, including various methods of data collection and analyzing and options for feeding-back measurement results to healthcare providers and facilities.

The decision of the G-BA to include the clinical domain CRC in the nationwide mandatory SQG program [24] reflects the necessity to provide information of the quality of CRC care as one of the most prevalent cancer entities nationwide [28]. In large international studies concerning cancer survival, it has been reported that data delivered form Germany covered only one to four percent of the national population [29] and the ‘international Agency for Research on Cancer’ assessed German cancer incidence rates as not valid [1].

### Quality indicator development

Indicators were developed using the ‘RAND/UCLA Appropriateness Method’ [26] that systematically combines scientific evidence and expert opinion and is proven to be a scientific sound method of indicator development [7]. Although there were disagreements between various disciplines within the multidisciplinary panel, it was possible to agree a final set of 52 indicators out of 210 candidate indicators presented to the panel. As demonstrated in previous literature, the panel composition of multidisciplinary medical professionals and patient representatives stimulated interaction during the consensus meetings and led to a more comprehensive set of indicators [17], [30].

As most quality indicators identified in the systematic search were developed in other countries, they could not be transferred directly between countries but had to be adapted to the German healthcare system and to the recommendations of the German S3-guideline on CRC during the panel process [31], [32]. As candidate indicators were presented in templates that included (where available) the underlying evidence of the indicator, indicators that were supported by high-level evidence-based guideline recommendations, were generally agreed unanimously by the panel members. The various medical disciplines involved in the care process of CRC were addressed comprehensively in the final set of indicators. However, clinical oncologists complained of the imbalance between the number of indicators representing surgery compared to chemotherapy and claimed a broader focus on chemotherapy indicators. Although chemotherapy is an essential component of multimodal therapy regimes for many patients with CRC, surgical therapy as a curative approach is related to a broader patient sample (denominator): According to the German multi-center study, over 90% of CRC-patients receive surgical treatment [33]. Measurement of chemotherapy indicators is more challenging as more quality information is needed to define the appropriate sample (denominator), as chemotherapy is suitable for only a portion of all patients with CRC and additionally, some of these patients are either unable to tolerate chemotherapy or refuse it. Even more information is needed for measuring the application of special chemotherapy agents or to reflect technical measures describing details of chemotherapy administration within a variety of chemotherapy-protocols and a variety of individual response. These limits led to the conclusion that the measurement of such indicators is not feasible [34].

It has been questioned whether participants of indicator-rating panels, usually expert clinicians, are qualified to rate the feasibility of indicators addressing operational issues of indicator implementation [35]. It seems to be difficult for panelists to assess the time and effort of data collection procedures necessary to operationalize an indicator [35]. Assessment of feasibility may be beyond the scope of clinical experts, as these are generally not experts for data collection and analyzes [8]. Therefore, ratings of experts can only provide a first appraisal concerning the feasibility of indicators, that has to be confirmed by data collection specialists and tested in practice using a validated testing protocol [20], [21].

Within the SQG program, special emphasis was placed on patients' perspectives, resulting in the participation of two patient-representatives in the multidisciplinary panel and the development of seven indicators reflecting patients' perspectives in particular. This was quite innovative, as it has been shown that patient participation during indicator development is extremely uncommon [19]. However, as patients' perspectives of quality assessment and medically based measures of quality may be different from each other [36], the inclusion of two patient representatives may not be sufficient to reflect patients' view comprehensively. As similar problems were observed in other SQG procedures, separate focus groups with patients will be established in future SQG procedures to supplement SQG-program methods [24], [25].

### Indicator data specification

In comparison to the role of panel ratings in identifying consensus and developing valid indicators, it is more challenging to specify how to measure agreed quality indicators [17]; particularly when measurement requires the combination of data sources from different healthcare sectors with variable data availability. Remuneration systems differ considerably between in- and outpatient settings. Minor problems were caused by inpatient data collection, where quality information could be derived from routine data including coded information on diagnoses based on the International classification of Diseases (ICD-10 GM) and also coded diagnostic and therapeutic procedures (OPS-codes). In the German ambulatory sector, however, OPS-codes were not available. Information on diagnoses (ICD-10 GM) and procedures (fee schedule items) could be derived from information systems that are used for clinical and administrative purposes by healthcare providers [25].

Quality measurement of follow-up procedures is resource intensive. First experiences in Germany concerning follow-up measurement were made with liver transplant donees whose follow up rates were only 67.5% [37]. In our study, it was proposed to use administrative data from sickness funds as the source for information on patients' vital status. As about ninety percent of the German population is covered by the statutory health insurance system (SHI), these data represent an innovative method for measuring risk adjusted long-term outcomes [37], [38].

Practice testing prior to usage of quality indicators is an important step to assess them against the required attributes of quality indicators such as validity, reliability, feasibility or sensibility to change [7]. As demonstrated in previous literature, only 10 to 20% of quality indicators developed for different clinical conditions have been measured during practice tests [8]. Although protocols for indicator practice testing are available [20], [21], technical specifications of measurement are sparse; even though this is an important step in preparing practice tests [39]. This includes also taking confounding factors into account due to case mix in hospitals and socio-demographic variables [8]. Adjustment for socio-demographic variables and case mix is very important for reliable interpretation of indicator results. Otherwise treatment of high-risk groups may be avoided by health care providers [40].

Although we were able to specify procedures for operationalizing all 52 indicators in the final set, with a preference for using routinely collected administrative and clinical data, a large amount of data remained to be collected by additional data recording for quality assurance purposes. This was considered by the AQUA-institute to reduce the feasibility of indicator implementation in routine settings. To reduce documentation efforts, alternative designs for data collection were described such as peer review audits. This method was also suggested to be superior concerning reliability, as complication rates for instance should not depend on coding of the treating physician because this is prone for manipulation. The implementation of new specific process codes (OPS-codes or fee schedule items) was proposed as another alternative data collection method that has to be considered and decided by the G-BA.

### Incorporation of results in established quality improvement strategies

Measurement of quality indicators is not an end in itself; it is the basis for developing and evaluating quality improvement strategies. In Germany, several quality initiatives are established to improve the quality of cancer care: The Ministry of Health established the National Cancer Plan in 2009 with the main focus on harmonizing treatment across the 16 disparate states [41]. Other quality improvement initiatives on CRC care were focused mainly on the hospital sector [25] or on colorectal surgery [33]. The challenge is now to integrate the SQG-program into existing initiatives, for instance the national cancer registries, to avoid redundant data entry. Therefore, working groups have been established to harmonize interests of various stakeholders and to discuss requirements with the G-BA.

### Strengths and limitations

The strength of the ‘RAND/UCLA Appropriateness Method’ is the combination of evidence from literature and experts' opinions that enables to provide a set of well-founded quality indicators [7]. The multidisciplinary composition of the panel and the intensive discussion during the panel meetings resulted in a set of indicators reflecting the entire pathway of care [30]. The nationwide, politically supported SQG-program has the potential to provide nationwide valid quality data on colorectal cancer and to link ambulatory and hospital services. Additionally, the methods developed in this study can provide case mix adjusted quality information protecting physicians and hospitals from an unjust appraisal of their performance.

Limitations of this study are that the harmonization of data entry and agreement with the G-BA are time consuming processes that will delay the further development of these indicators such as feasibility field testing and the roll out of the program.

## Conclusions

In Germany, a set of 52 quality indicators, covering all relevant aspects of the CRC care processes has been developed that address cross-sectoral interfaces. Combining different sectors and sources of health care in quality assessment is an innovative and challenging approach but reflects better the reality of patient experiences of CRC, rather than sets of indicators that address individual sectors (ambulatory or hospital) in isolation. It reflects the interdisciplinary coordination that embodies CRC-care and will help address quality improvement across health care interfaces.

## Supporting Information

Table S1 Description of quality indicators for colorectal cancer care.
(DOCX)

Table S2 Preliminary search.
(DOCX)

Table S3 Systematic literature search – search terms and procedures.
(DOCX)

Table S4 Systematic literature search – hits.
(DOCX)

Table S5 Systematic literature search - inclusion and exclusion criteria.
(DOCX)

Table S6 Systematic literature search – included papers.
(DOCX)

Table S7 Search of indicator databases.
(DOCX)
