# Supplementary material for: Evaluating the Quality of Colorectal Cancer Care across the Interface of Healthcare Sectors
Source: PLoS One. 2013 May 1;8(5):e60947. doi: 10.1371/journal.pone.0060947 (PMC3641026; doi:10.1371/journal.pone.0060947)
Supplement: Table S1 — Description of quality indicators for colorectal cancer care. (DOCX) [file pone.0060947.s001.docx]

**Table S1: Description of quality indicators for colorectal cancer care**

| Indicator 1 | | | Availability and constitution of multidisciplinary tumor boards/ ambulatory multidisciplinary teams | | |  |  |  |
| --- | --- | --- | --- | --- | --- | --- | --- | --- |
| Numerator | | | Number of institutions/ ambulatory multidisciplinary teams, that provide a tumor board with the following structural conditions:  The tumor board/ambulatory multidisciplinary team should discuss 50 cases per year and decide on treatment planning.  The core team members should be designated and include: a visceral surgeon, a radiation oncologist, a (diagnostic) radiologist, a gastroenterologist, a medical oncologist, a pathologist. | | |  |  |  |
| Denominator | | | All institutions/ ambulatory multidisciplinary teams | | |  |  |  |
| Indicator 2 | | | Pre-therapeutic assessment of CRC-patients by tumor boards/ambulatory multidisciplinary teams | | |  |  |  |
| Numerator | | | Number of patients, which had an pre-therapeutic assessment by a tumor board/ambulatory multidisciplinary team | | |  |  |  |
| Denominator | | | All patients with:  1. RC or  2. Metastasizing CC or  3. CRC and loco-regional recurrence | | |  |  |  |
| Indicator 3 | | | Tumor board/ambulatory multidisciplinary team with expertise in metastatic surgery | | |  |  |  |
| Numerator | | | Number of tumor boards/ambulatory multidisciplinary teams with expertise (hepato-biliary surgeons, oncologists) and technical qualification for surgery of liver metastases | | |  |  |  |
| Denominator | | | All institutions/ambulatory multidisciplinary teams with tumor board | | |  |  |  |
| Indicator 4 | | | Availability and content of a preoperative colonoscopy report | | |  |  |  |
| Numerator | | | Number of patients, which had a colonoscopy report preoperatively, which included the following quality criteria:  Completeness of the examinations  Localization of the tumor  Description of the tumor (for example: small, medium, big, circumferential)  Measured size of the tumor | | |  |  |  |
| Denominator | | | All patients with CRC, which had a resection of the primary tumor. | | |  |  |  |
| Indicator 5 | | | Colonoscopy reports with documentation of specific quality aspects | | |  |  |  |
| Numerator | | | Number of colonoscopy reports, which content the following aspects completely:  Pre-procedural risk estimation  Completeness of colonoscopy  Quality of bowel preparation  Complete description of all located polyps, including localization of each polyp, size, number and macroscopic morphology  Recommendation for follow-up | | |  |  |  |
| Denominator | | | Colonoscopy reports of patients with CRC of an institution (random sample) | | |  |  |  |
| Indicator 6 | | | Pre-therapeutic availability of a histo-pathologic diagnosis (tumor biopsy) | | |  |  |  |
| Numerator | | | Number of patients, in which the diagnosis was confirmed by pre-therapeutic biopsy | | |  |  |  |
| Denominator | | | All patients with CRC, who received a primary therapy (resection of the primary tumor or a neo-adjuvant radio(chemo)therapy) | | |  |  |  |
| Indicator 7 | | | Pre-therapeutic liver imaging in patients with CRC | | |  |  |  |
| Numerator | | | Number of patients, who had a pre-therapeutic liver imaging with ultrasound or CT or MRI | | |  |  |  |
| Denominator | | | All patients with CRC, who received a primary therapy (resection of the primary tumor or a neo-adjuvant radio(chemo)therapy) | | |  |  |  |
| Indicator 8 | | | Pre-therapeutic rigid rectoscopy in RC-patients | | |  |  |  |
| Numerator | | | Number of patients, who had a pre-therapeutic rigid rectoscopy to define the distance of the lower tumor margin to the anocutaneous line (description in cm) | | |  |  |  |
| Denominator | | | All patients with RC, who received a primary therapy (resection of the primary tumor or a neoadjuvant radio(chemo)therapy) | | |  |  |  |
| Indicator 9 | | | Pre-therapeutic staging using cTNM-categories in RC-patients | | |  |  |  |
| Numerator | | | Number of patients with pre-therapeutic assessment:  Depth of tumor invasion (cT),  Peri-rectal lymph nodes (cN) and  Diagnostic of metastases (cM) | | |  |  |  |
| Denominator | | | All patients with CRC, who received a primary therapy (resection of the primary tumor or a neo-adjuvant radio(chemo)therapy) | | |  |  |  |
| Indicator 10 | | | Pre-therapeutic pelvis imaging using multi-slice CT or high-resolution MRI in RC-patients | | |  |  |  |
| Numerator | | | Number of patients, who had pre-therapeutic imaging of the pelvis with multi-slice CT or high-resolution MRI | | |  |  |  |
| Denominator | | | All patients with CRC, who received a primary therapy (resection of the primary tumor or a neo-adjuvant radio(chemo)therapy) | | |  |  |  |
| Indicator 11 | | | Pre-therapeutic imaging of liver and lungs using CT or MRI in CRC-patients with liver metastases | | |  |  |  |
| Numerator | | | Number of patients, who had a pre-therapeutic CT or MRI (PET-CT only with specific indications) of the liver or the lungs | | |  |  |  |
| Denominator | | | All patients with CRC and CRC-liver metastasis | | |  |  |  |
| Indicator 12 | | | Pre-operative assessment of bowel, urinary and sexual function in RC-patients | | |  |  |  |
| Numerator | | | Scores (with EORTC-QLQ-CR 29 questionnaire) of the functional status of the preoperative bowel, urinary and sexual function | | |  |  |  |
| Denominator | | | All patients with RC, who have had surgery and were randomly selected for participation in the patient survey | | |  |  |  |
| Indicator 13 | | | Assessment of Bethesda-criteria in patients with CRC | | | | |  |
| Numerator | | | Number of patients, who had assessment and documentation of the Bethesda-criteria | | | | |  |
| Denominator | | | All patients with CRC, who had resection of the primary tumor | | | | |  |
| Indicator 14 | | | Preoperative stoma education where appropriate | | |  |  |  |
| Numerator | | | Number of patients who had preoperative counseling regarding a planned stoma | | |  |  |  |
| Denominator | | | All patients with CRC, who had a stoma-surgery | | |  |  |  |
| Indicator 15 | | | Preoperative marking of stoma-localization in surgery of CRC | | |  |  |  |
| Numerator | | | Number of patients, who had preoperatively marked the localization of the stomain a sitting and standing position | | |  |  |  |
| Denominator | | | All patients with CRC, who had a stoma-surgery | | |  |  |  |
| Indicator 16 | | | Neoadjuvant radio(chemo)therapy in RC-patients | | |  |  |  |
| Numerator | | | Number of patients, who had a neo-adjuvant radio(chemo)therapy. | | |  |  |  |
| Denominator | | | All patients with RC of the mid and low third rectum and TNM-categories  cT3, 4/cM0 and/or  cN1, 2/cM0,  who had surgery | | |  |  |  |
| Indicator 17 | | | Radiotherapy in line with quality standards of the German Society of Radiation Oncology (DEGRO) in RC-patients | | |  |  |  |
| Numerator | | | Number of patients with RC, who had radiotherapy according to the following quality criteria of the German Association of Radio-oncology (Deutsche Gesellschaft für Radioonkologie, DEGRO):  1. Availability of a 3-D-plan with CT and a maximum of 5mm layer thickness  2. Application of a minimum of 3 radiation fields, which get radiated daily  3. Discontinuity of the radiation (course) less than 5 days  4. Maintaining of the dosage of 5x5 = 25 Gy during short-time radiation  5. Maintaining of a total dose of a minimum of 45 Gy during long-time radiation  6. Application of chemotherapy simultanously to radiotherapy during long-time radiation. | | |  |  |  |
| Denominator | | | All patients with RC, who received radiotherapy | | |  |  |  |
| Indicator 18 | | | Antibiotic prophylaxis before CRC-surgery | | |  |  |  |
| Numerator | | | Number of patients, who received antibiotic prophylaxis before surgical tumor resection | | |  |  |  |
| Denominator | | | All patients with CRC, who had surgery | | |  |  |  |
| Indicator 19 | | | En bloc resection in case of tumor adherence to other organs | | |  |  |  |
| Numerator | | | Number of patients, who had en bloc resection | | |  |  |  |
| Denominator | | | All patients with CRC, of whom tumor adherence to other organs was found during surgery | | |  |  |  |
| Indicator 20 | | | Intra-operative exploration of liver and peritoneal lining | | |  |  |  |
| Numerator | | | Number of patients, who had intra-operatively an exploration of liver and peritoneal lining | | |  |  |  |
| Denominator | | | All patients with CRC, who had surgery | | |  |  |  |
| Indicator 21 | | | Intraoperative local dissemination of tumor cells | | |  |  |  |
| Numerator | | | Number of patients, in which the pathologist had documented an intra-operative local dissemination of tumorcells | | |  |  |  |
| Denominator | | | All patients with CRC and primary radical resection of the tumor (R0-resection) | | |  |  |  |
| Indicator 22 | | | Total/partial mesorectal excision (TME/PME)in RC-patients | | |  |  |  |
| Numerator | | | Number of patients, who received a TME (if localization of tumor was in the lower or middle third of the rectum) or a PME (if localization of tumor was in the high third of the rectum) | | |  |  |  |
| Denominator | | | All patients with RC, who had surgery. | | |  |  |  |
| Indicator 23 | | | Abdominal perineal resection (APR) in RC-patients | | |  |  |  |
| Numerator | | | Number of patients, who received an abdominal perineal resection | | |  |  |  |
| Denominator | | | All patients with RC, who had surgery | | |  |  |  |
| Indicator 24 | | | Major anastomotic leakage after elective CRC-surgery | | | |  |  |
| Numerator | | | Number of patients with post-surgical interventions due to a clinically manifest anastomotic leakage | | | |  |  |
| Denominator | | | All patients with CRC, who had elective surgery | | | |  |  |
| Indicator 25 | | | Surgical re- interventions after CRC-surgery | | | |  |  |
| Numerator | | | Number of patients with abdominal re-interventions due to complications during the acute inpatient care | | | |  |  |
| Denominator | | | All patients with CRC, who had resection of the primary tumor | | | |  |  |
| Indicator 26 | | | Examination of least 12 lymph nodes | | | |  |  |
| Numerator | | | Number of patients with at least 12 removed and patho-histologically examined lymph nodes | | | |  |  |
| Denominator | | | All patients with CRC, who had resection of the primary tumor. | | | |  |  |
| Indicator 27 | | | Rate of local R0-resection in patients with CRC | |  |  |  |  |
| Numerator | | | Number of patients with local R0-resection | |  |  |  |  |
| Denominator | | | All patients with CRC, who had resection of the primary tumor | |  |  |  |  |
| Indicator 28 | | | Rate of pT1 carcinoma in CRC-patients | | |  |  |  |
| Numerator | | | Number of patients with pT1 carcinoma | | |  |  |  |
| Denominator | | | All patients with CRC, who had resection of the primary tumor | | |  |  |  |
| Indicator 29 | | | Liver- and lung-metastasectomy in patients with stage IV CRC | | |  |  |  |
| Numerator | | | Number of patients who had metastasectomy (of liver or lung) | | |  |  |  |
| Denominator | | | All patients with CRC and metastasis of liver or lung | | |  |  |  |
| Indicator 30 | | | Documentation of distal tumor-free margin in RC-patients | | |  |  |  |
| Numerator | | | Number of patients with documentation of the distance of distal tumor margin to the distal resection margin in mm and of the distance of the tumor to circumferential meso-rectal resection margin in mm | | |  |  |  |
| Denominator | | | All patients with RC, who had resection of the primary tumor with TME or PME | | |  |  |  |
| Indicator 31 | | | Mesorectal CRM-positive (CRM < 1mm) radical surgical resection in RC-patients | | |  |  |  |
| Numerator | | | Number of patients, who had a specimen with positive circumferential margin (CRM < 1mm). | | |  |  |  |
| Denominator | | | All patients with RC, who had a radical resection of the tumor in curative intention. | | |  |  |  |
| Indicator 32 | | | Quality of Total Mesorectal Excision (TME) | | | | | |
| Numerator | | | Number of patients with good or moderate quality of TME | | | | | |
| Denominator | | | All patients with RC, who had TME | | | | | |
| Indicator 33 | | | Pathology reports following quality standards of the German Society of Pathology | | | |  |  |
| Numerator | | | Number of pathology reports, which include the following criteria completely.  Localization  Type of tumor according to WHO classification  Depth of tumor invasion (pT-classification)  Status of regional lymph nodes (pN-classification)  Number of examined lymph nodes  Number of affected lymph nodes  Grading  Distance to resection margins  R-classification  Invasion of lymph-/ blood vessels  In RC-specimens additionally  Quality of TME  Grade of tumor-regression in case of neo-adjuvant therapy (if applicable) | | | |  |  |
| Denominator | | | All pathology reports of patients with CRC and surgical tumor-resection | | | |  |  |
| Indicator 34 | | | Postoperative pain assessment after surgery of CRC | | | |  |  |
| This indicator was excluded after the panel ratings because it was already included in a general part of the patient survey | | | | | | |  |  |
| Indicator 35 | | | Post-operative assessment of bowel, urinary and sexual function in RC-patients | | | |  |  |
| Numerator | | | Changes of scores (EORTC-QLQ-CR 29 questionnaire) of the functional status of the prost-operative bowel, urinary and sexual function in comparison to the preoperative score (see indicator 12) | | | |  |  |
| Denominator | | | All patients with RC, who have had surgery and were randomly selected for participation in the patient survey | | | |  |  |
| Indicator 36 | | | Providing of information and instructions about stoma management in patients with stoma | | | |  |  |
| Numerator | | | Number of patients with stoma, who received information about stoma care before discharge including the following aspects:  Instructions about stoma care  A contact person (stoma therapist) for stoma care | | | |  |  |
| Denominator | | | All patients with CRC, who have had surgery and were randomly selected for participation in the patient survey | | | |  |  |
| Indicator 37 | | | Adjuvant chemotherapy in patients with stage III CC | | | |  |  |
| Numerator | | | Number of patients, who received adjuvant chemotherapy | | | |  |  |
| Denominator | | | All patients with CC UICC-stage III, who had R0-resection of the primary tumor | | | |  |  |
| Indicator 38 | | Time interval between surgery and starting adjuvant chemotherapy in patients with stage III CC | |  |  |  |  |  |
| Numerator | | Number of patients with a time period of less than 6 weeks until start of an adjuvant chemotherapy. | |  |  |  |  |  |
| Denominator | | All patients with CC UICC-stage III, who had R0-resection of the primary tumor. | |  |  |  |  |  |
| Indicator 39 | | Documentation of chemotherapy treatment summary in medical records and passing on this information to the patient and to the physician providing surveillance | |  |  |  |  |  |
| Numerator | | Number of patients, of whose the following aspects were documented in the medical chart:  A summary of the chemotherapy treatment,  Its transition to the patient and  Its transition to the physician(s) providing continuing care. | |  |  |  |  |  |
| Denominator | | All patients with CRC, who received chemotherapy | |  |  |  |  |  |
| Indicator 40 | | Delivery of a written plan for pain management in CRC-patients where appropriate | |  |  |  |  |  |
| Numerator | | Number of patients, who received a plan for pain management | |  |  |  |  |  |
| Denominator | | All patients with CRC, who have had surgery and were randomly selected for participation in the patient survey | |  |  |  |  |  |
| Indicator 41 | | Examination of microsatellite-instability in CRC-patients younger than 50 years | |  |  |  |  |  |
| Numerator | | Number of patients, in which an examination for microsatellite-instability was performed | |  |  |  |  |  |
| Denominator | | All patients with CRC under 50 years of age, who had a resection of the primary tumor | |  |  |  |  |  |
| Indicator 42 | | Postoperative colonoscopy within 6 months in patients with incomplete preoperative colonoscopy | |  |  |  |  |  |
| Numerator | | Number of patients, who received a complete colonoscopy within 6 months postoperatively | |  |  |  |  |  |
| Denominator | | All patients with CRC, who had surgery and who did not have complete colonoscopy preoperatively | |  |  |  |  |  |
| Indicator 43 | | Postoperative surveillance as recommended in the German evidence-based guideline | | | |  |  |  |
| Numerator | | Number of patients, who undergo surveillance | | | |  |  |  |
| Denominator | | All patients with stage II and III UICC after R0-resection | | | |  |  |  |
| Indicator 44 | | Sharing the decision with the patient regarding therapeutic procedures | | | |  |  |  |
| Numerator | | Number of patients, who claim they had been involved by therapists in the decision making process regarding therapeutic procedures | | | |  |  |  |
| Denominator | | All patients with CRC, who have had surgery and were randomly selected for participation in the patient survey | | | |  |  |  |
| Indicator 45 | | Opportunities to ask the specialists questions | | | |  |  |  |
| Numerator | | Number of patients, who claim they had sufficient opportunities to ask questions | | | |  |  |  |
| Denominator | | All patients with CRC, who had a resection of the primary tumor and who participated in the patients’ survey | | | |  |  |  |
| Indicator 46 | | The patient is offered contact with a companion in distress | | | |  |  |  |
| Numerator | | Number of patients, who claim they had been offered a contact person for distress | | | |  |  |  |
| Denominator | | All patients with CRC, who have had surgery and were randomly selected for participation in the patient survey | | | |  |  |  |
| Indicator 47 | | The patient knows, which activities are allowed at home | | | |  |  |  |
| Numerator | | Number of patients, who claim they had been told, which activities are allowed at home | | | |  |  |  |
| Denominator | | All patients with CRC, who have had surgery and were randomly selected for participation in the patient survey | | | |  |  |  |
| Indicator 48 | | The patient knows, which side effects or late complications to be aware of at home | | | |  |  |  |
| Numerator | | Number of patients, who claim they had been told, which side effects or late complications to be aware of at home | | | |  |  |  |
| Denominator | | All patients with CRC, who have had surgery and were randomly selected for participation in the patient survey | | | |  |  |  |
| Indicator 49 | | The patient knows, when to contact physicians providing continuing care | | | |  |  |  |
| Numerator | | Number of patients, who claim they had been told, when to contact their general practitioner or a specialist. | | | |  |  |  |
| Denominator | | All patients with CRC, who have had surgery and were randomly selected for participation in the patient survey | | | |  |  |  |
| Indicator 50 | 5-year overall survival in CRC-patients | | | | |  |  |  |
| Numerator | Number of patients, who survived at least 5 years | | | | |  |  |  |
| Denominator | All patients with CRC, who have had surgery and were randomly selected for participation in the patient survey | | | | |  |  |  |
| Indicator 51 | | 5-year local recurrence rate in RC-patients | | | |  |  |  |
| Numerator | | Number of patients, who had surgery of a local recurrence. | | | |  |  |  |
| Denominator | | All patients with RC UICC-stage I-III, who had R0-resection of the primary tumor. | | | |  |  |  |
| Indicator 52 | 30-day-mortality rate after primary CRC-surgery | | | | |  |  |  |
| Numerator | | Number of patients, who died within a time period of 30 days after surgery. | | | |  |  |  |
| Denominator | | All patients with CRC, who had resection of the primary tumor. | | | |  |  |  |
| Indicator 53 | | Assessment of quality of life with a specific instrument in CRC-patients | | | |  |  |  |
| Numerator | | Number of patients with CRC, in which the quality of life was assessed with a specific survey instrument (EORTC QLQ-C30) | | | |  |  |  |
| Denominator | | All patients with CRC, who have had surgery and were randomly selected for participation in the patient survey | | | |  |  |  |
