# Supplementary material for: Evaluating the Quality of Colorectal Cancer Care across the Interface of Healthcare Sectors
Source: PLoS One. 2013 May 1;8(5):e60947. doi: 10.1371/journal.pone.0060947 (PMC3641026; doi:10.1371/journal.pone.0060947)
Supplement: Table S2 — Preliminary search. (DOCX) [file pone.0060947.s002.docx]

**Table S2: Preliminary search**

| **Data sources and included papers** |
| --- |
| **1. Cochrane Database: Reviews of the “Cochrane colorectal cancer group”** |
| 1. Amato A, Pescatori M. Perioperative blood transfusions for the recurrence of colorectal cancer. Cochrane Database Syst Rev 2006.  2. Best L, Simmonds P, Baughan C, Buchanan R, Davis C, Fentiman I, et al. Collaboration Colorectal Meta-analysis. Palliative chemotherapy for advanced or metastatic colorectal cancer. Cochrane Database Syst Rev 2000.  3. Breukink S, Pierie JP, Wiggers T. Laparascopic versus open total mesorectal excision for rectal cancer. Cochrane Database Syst Rev 2006.  4. Chionh F, Campbell A, Sukumaran S, Price T, Tebbutt N. Oral versus intravenous fluoropyramidines for colorectal cancer (protocol). Cochrane Database Syst Rev 2010.  5. Choy PYG, Bissett IP, Docherty JG, Parry BR, Merrie A. Stapled versus handsewn methods for ileocolic anastomoses. Cochrane Database Syst Rev 2007.  6. de Jesus EC, Karliczek A, Matos D, Castro AA, Atallah AN. Prophylactic anastomotic drainage for colorectal surgery. Cochrane Database Syst Rev 2004.  7. De Salvo GL, Gava C, Lise M, Pucciarelli S. Curative Surgery for obstruction from primary left colorectal carcinoma: primary or staged resection? Cochrane Database of Syst Rev 2006.  8. Figueredo A, Coombes ME, Mukherjee S. Adjuvant Therapy for completely resected stage II colon cancer. Cochrane Database Syst Rev 2008.  9. Gao F, Cao Y, Liao C, Mo Z, Tan A. Sequential versus combination chemotherapy for advanced colorectal cancer (protocol). Cochrane Database Syst Rev 2010.  10. Guenaga KF, Lustosa SA, Saad SS, Saconato H, Matos D. Ileostomy or colostomy for temporary decompression of colorectal anastomosis. Cochrane Database Syst Rev 2007.  11. Gulla N, Cirocchi R, Boselli C, Abraha I, Trastulli S, Montedori A, et al. Radiofrequency ablation in the treatment of liver metastases from colorectal cancer (Protocol). Cochrane Database Syst Rev 2010.  12. Hewitt J, McCarthy K. Neoadjuvant chemoradiation for non-metastatic locally advanced rectal cancer (Protocol). Cochrane Database Syst Rev 2010.  13. Iversen LH, Wille Jorgensen P, Borowski D, Archampong D. Workload and surgeon's specialty for outcome after colorectal cancer surgery (protocol). Cochrane Database Syst Rev 2010.  14. Jeffery M, Hickey BE, Hider PN. Follow-up strategies for patient streated for non-metastatic colorectal cancer. Cochrane Database Syst Rev 2007.  15. Kuhry E, Schwenk W, Gaupset R, Romild U, Bonjer HF. Long-term results of laparoscopic colorectal cancer resection. Cochrane Database Syst Rev 2008.  16. Matos D, Atallah AN, Castro AA, Silva Lustosa SA. Stapled versus handsewn methods for colorectal anastomisis surgery. Cochrane Database Syst Rev s 2001.  17. Magaji BA, Roslani AC, Chee Wei L, Moy FM, Buckley BS. Closed versus open approach in laparascopic colorectal surgery (protocol). Cochrane Database Syst Rev 2008.  18. Moloo H, Haggar F, Coyle D, Hutton B, Duhaime S, Mamazza J, et al. Hand assisted laparascopic surgery versus conventional laparascopy for colorectal surgery. Cochrane Database Syst Rev 2010.  19. Nelson RL, Freels S. Hepatic artery adjuvant chemotherapy for patients having resection or ablation of colorectal cancer metastatic to the liver. Cochrane Database Syst Rev 2006.  20. Nienhuijs SW, Kuhry E, de Hingh I, Lemmens V, Marvik R. Transanal endoscopic microsurgery (TEM) compared to radical surgery for rectal cancer (Protocol). Cochrane Database of Systematic Reviews 2010.  21. Ng RCH, Fitzharris BM, Hider PN, Jeffery M. Chemotherapy with platinum compounds for metastatic colorectal cancer (Protocol). Cochrane Database Syst Rev 2003.  22. Pachler J, Wille Jorgensen P. Quality of life after rectal resection for cancer, with or without permanent colostomy. [Review] [58 refs][Update of Cochrane Database Syst Rev. 2004;(3):CD004323; PMID: 15266529]. Cochrane Database Syst Rev 2005.  23. Schwenk W, Haase O, Neudecker J, Mller JM. Short term benefits for laparoscopic colorectal resection. Cochrane Database Syst Rev 2005.  24. Spanjersberg WR, Reurings J, van Laarhoven C. Fast track surgery versus conventional recovery strategies for colorectal surgery (protocol). Cochrane Database Syst Rev 2009.  25. Townsend A, Price T, Karapetis C. Selective internal radiation therapy for liver metastases from colorectal cancer. Cochrane Database Syst Rev 2009.  26. Wagner A, Arnold D, Grothey A, Haerting J, Unverzagt S. Antiangiogenic therapies for metastatic colorectal cancer. Cochrane Database Syst Rev 2009.  27. Wong RKS, Tandan V, De Silva S, Figueredo A. Pre-operative radiotherapy and curative surgery for the management of localized rectal carcinoma. Cochrane Database Syst Rev 2007.  28. Wu T, Munro AJ, Guanjian L, Liu GJ. Chinese medical herbs for chemotherapy side effects in colorectal cancer patients. Cochrane Database Syst Rev 2005. |

| **Data sources and included papers** | | |
| --- | --- | --- |
| **2. : Guideline Databases**  **- AWMF (Arbeitsgemeinschaft der Wissenschaftlichen Medizinischen Fachgesellschaften)**  **- National Guideline Clearinghouse** | | |
| N = 1 German S3-Leitlinie (highest level of evidence)  S3-Leitlinie „Kolorektales Karzinom“ 2004/2008. 2008 (Schmiegel et al. 2008)  N = 3 Supplementary German guidelines  S1-Leitlinie (AWMF) Supportive Maßnahmen in der Radioonkologie 2006  S2-Leitlinie (AWMF) Leitlinie für die Begutachtung von Schmerzen 2007  Deutsche Gesellschaft für Allgemeinmedizin: Pflegende Angehörige 2005  N = 44 International guidelines and **practical guidelines**  ***Screening:***  American Cancer Society, US Multi-Society Task Force on Colorectal Cancer, American College of Radiology. Screening and surveillance for the early detection of colorectal cancer and adenomatous polyps. 2006.  American College of Gastroenterology. Guidelines for Colorectal Cancer Screening. 2008  American Society for Gastrointestinal Endoscoopy (ASGE). Colorectal cancer screening and surveillance. 2006  Canadian Association of Gastroenerology and the Canadian Digestive Health Foundation. Guidelines for colon cancer screening. 2004  Canadian Task Force on Preventive Health Care: Screening strategies for colorectal cancer: Systematic review & recommendations. 2001  Gastrointestinal Consortium Panel. Colorectal cancer screening and surveillance: clinical guidelines and rationale-Update based on new evidence. 2003  Institute for Clinical Systems Improvement. Colorectal Cancer Screening. 2008  Kaiser Permanente Care Management Institute. Colorectal cancer screening clinical practice guideline. 2008  Michigan Quality Improvement Consortium. Adult preventive services (Ages 18-49). 2008  Michigan Quality Improvement Consortium. Adult Preventive Services (Ages 50-65+)  National Committee for Quality Assurance. Colorectal cancer screening. 2004  Society of Gastroenterology Nurses and Associates (SGNA). Performance of flexible sigmoidoscopy by registered nurses for the purpose of colorectal cancer screening. 2003  U.S. Preventive Services Task Force (USPSTF). Screening for colorectal cancer: U.S. Preventive Services Task Force recommendation statement. 2008  U.S. Preventive Services Task Force. Routine aspirin or nonsteroidal anti-inflammatory drugs for the primary prevention of CRC. 2007  ***Colorectal Cancer*:**  Scottish Intercollegiate Guidelines Network (SIGN). Management of Colorectal Cancer - A national clinical guideline. 2003  The Association of Coloproctology of Great Britain and Ireland. Guidelines for the Management of Colorectal Cancer 3rd edition. 2007  The Cancer Council Australia/Australian Cancer Network. Clinical Practice Guidelines for the prevention, early detection and management of colorectal cancer. 2005  ***Colon Cancer:***  Association of Comprehensive Cancer Centres (NL). Colon Cancer. 2008  European Society for Medical Oncology (ESMO). Minimal clinical recommendations for diagnosis, adjuvant treatment and follow-up of colon cancer. 2005  National Comprehensive Cancer Network. Colon cancer clinical practice guidelines in oncology. 2005  ***Rectum Cancer****:*  Association of Comprehensive Cancer Centres (NL). Rectal cancer. 2008  European Society for Medical Oncology (ESMO). Minimal clinical recommendations for diagnosis, treatment and follow-up of rectal cancer. 2005  National Comprehensive Cancer Network. Rectal cancer clinical practice guidelines in oncology. 2005 | | |
| **Data sources and included papers** | | |
| **2. : Guideline Databases**  **- AWMF (Arbeitsgemeinschaft der Wissenschaftlichen Medizinischen Fachgesellschaften)**  **- National Guideline Clearinghouse** | | |
| ***Metastasized Colorectal Cancer:***  European Society for Medical Oncology (ESMO). Minimal clinical recommendations for diagnosis, treatment and follow-up of advanced colorectal cancer. 2005  National Institute for Clinical Excellence (NHS). Radiofrequency ablation for colorectal liver metastases. 2009  National Institute for Clinical Excellence (NHS). Selective internal radiation therapy for colorectal metastases in the liver. 2004  ***Risk groups:***  Evaluation of Genomic Applications in Practice and Prevention (EGAPP) Working Group. Genetic testing strategies in newly diagnosed individuals with colorectal cancer aimed at reducing morbidity and mortality from Lynch syndrome in relatives. 2009  ***Diagnostic procedures:***  American College of Radiology. ACR Appropriateness Criteria pretreatment staging of colorectal cancer. 2008  American Society of Clinical Oncology. 2000 update of recommendations for the use of tumor markers in breast and colorectal cancer: clinical practice guidelines. 2001  ***Endoskopy:***  American Society for Gastrointestinal Endoscoopy (ASGE). The role of endoscopy in the diagnosis, staging, and management of colorectal cancer. 2005  ***Surgery:***  National Cancer Institute Expert Panel. Guidelines 2000 for colon and rectal cancer surgery. 2001  National Institute for Health and Clinical Excellence (NICE). Laparascopic surgery for colorectal cancer. 2006  ***Pathology:***  Association of Directors of Anatomic and Surgical Pathology. Recommendations for the reporting of surgically resected specimens of colorectal carcinoma. 2008  ***Adjuvant chemotherapy****:*  American Society of Clinical Oncology. Recommendations on adjuvant chemotherapy for stage II colon cancer. 2004  Cancer Care Ontario Program. Adjuvant systemic chemotherapy for stage II and III colon cancer following complete resection: guideline recommendations. 2008  National Institute for Health and Clinical Excellence (NICE). Bevacizumab and cetuximab for the treatment of metastatic colorectal cancer. 2007  ***Radiotherapy:***  Cancer Care Ontario’s program in Evidence-based Care’s Gastrointestinal Cancer Disease Site Group. The use of preoperative radiotherapy in the management of patients with clinically respectable rectal cancer: a practice guideline. 2003  National Institute for Clinical Excellence (NHS). Preoperative high dose rate brachytherapy for rectal cancer. 2006  ***Surveillance:***  American Cancer Society, US Multi- Society Task Force on Colorectal Cancer. Guidelines for colonoscopy surveillance after cancer resection: A consensus update by the American Cancer Society and US Multi-Society Task Force on colorectal cancer. 2006  American Society of Clinical Oncology. Colorectal Cancer Surveillance: 2005 Update of an American Society of Clinical Oncology Practice Guideline. 2005  Association of Coloproctology for Great Britain and Ireland. Guidelines for follow-up after resection of colorectal cancer. 2002  Cancer Care Ontario Program. Follow-up of patients with curatively resected colorectal cancer. 2004  Gastrointestinal Cancer Disease Site Group of Cancer Care Ontario’s Program in Evidence-based Care. Follow-up of patients with curatively resected colorectal cancer: a practice guideline. 2003  The American Society of Colon and Rectal Surgeons. Practice Parameters for the surveillance and follow-up of patients with colon and rectal cancer. 2004 | | |
| **Data sources and included papers** | |  |
| **3. Health Technology Assessment (HTA)** |  |  |
| N = 1 (Quality indicators for colorectal cancer)  Pathwardan M et al. (Agency for Healthcare Research and Quality). Cancer care quality measures: diagnosis and treatment of colorectal cancer. 2006(Patwardhan et al. 2006) |  |  |
| **4. Other papers** | |  |
| 1. Baessler K, Kempkensteffen C. Validierung eines umfassenden Beckenboden-Fragebogens für Klinik, Praxis und Forschung. Gynakol Geburtshilfliche Rundsch 2009; 49:299-307.  2. Clasen S, Rempp H, Pereira PL. Metastasen des kolorektalen Karzinoms. Der Radiologe 2008; 48:1032-42.  3. Dietel M, Tannapfel A, Baretton G, Kreipe H, Kloor M, Gabbert H, et al. Molekularpathologische Analyse des K-RAS-Mutationsstatus beim metastasierten kolorektalen Karzinom. Ein Beispiel pr„diktiver Pathologie. Onkologe 2008; 14 405-8.  4. Dworak O, Keilholz L, Hoffmann A. Pathological features of rectal cancer after preoperative radiochemotherapy. International Journal of Colorectal Disease 1997; 12:19-23.  5. Gujral S, Conroy T, Fleissner C, Sezer O, King PM, Avery KNL, et al. Assessing quality of life in patients with colorectal cancer: An update of the EORTC quality of life questionnaire. European Journal of Cancer 2007; 43:1564-73.  6. Grundmann RT, Hermanek P, Merkel S, Germer CT, Hauss J, Henne-Bruns D, et al. Diagnostik und Therapie von Lebermetastasen kolorektaler Karzinome - Workflow. ZentralblChir 2008; 133:267-84.  7. Grothey A, Kellermann L, Schmoll HJ. Defizite in der Behandlung von Patienten mit kolorektalem Karzinom in Deutschland. Ergebnisse einer multizentrischen Dokumentation von Therapiealgorithmen. Medizinische Klinik 2002; 97:270-7.  8. Herschbach P. Behandlungsbedarf in der Psychoonkologie. Der Onkologe 2006; 12:41-7.  9. Hoelzel D, Schubert-Fritschle G, Engel J. Ergebnisse der interdisziplin„ren onkologischen Versorgung. Onkologe 2009; 15 1120-33.  10. Hofstaetter F, Hoelzel D. Was leisten Tumorregister fuer die Qualitaetssicherung in der Onkologie? Der Onkologe 2008; 14:1220-33.  11. Juchems MS, Aschoff AJ. Aktuelle Bildgebung bei Rektumkarzinom. Chirurg 2009; 80:274-80.  12. Kube R, Mroczkowski P, Steinert R, Sahm M, Schmidt U, Gastinger I, et al. Anastomoseninsuffizienzen nach Kolonkarzinomresektionen. Multiple Analyse der Risikofaktoren. Chirurg 2009; 80:1153-9.  13. Lemmens VE, van H, Janssen Heijnen ML, Vreugdenhil G, Repelaer v, Coebergh JW. Adjuvant treatment for elderly patients with stage III colon cancer in the southern Netherlands is affected by socioeconomic status, gender, and comorbidity. Annals of Oncology 2005; 16:767-72.  14. Link KH, Kornmann M, Bittner R, Koeckerling F, Arbogast R, Gastinger I, et al. Qualitaetsanforderungen zur Behandlung des Kolon- und Rektumkarzinoms. Der Chirurg 2010; 81:222-30.  15. Luo R, Giordano SH, Freeman JL, Zhang D, Goodwin JS. Referral to Medical Oncology: A Crucial Step in the Treatment of Older Patients with Stage III Colon Cancer. The Oncologist 2006; 11:1025-33.  16. Mahboubi A, Lejeune C, Coriat R, Binquet C, Bouvier AM, Bejean S, et al. Which patients with colorectal cancer are followed up by general practitioners? A population-based study. European Journal of Cancer Prevention 2007; 16:535-41.  17. Mantke R, Niepmann D, Gastinger I, Lippert H, Koch K, Quehl A. Kurative und diagnostische Resektionen an der Leber. Der Chirurg 2006; 77:1135-43.  18. Marusch F, Koch A, Schmidt U, Zippel R, Geissler S, Pross M, et al. Prospektive Multizenterstudien "Kolon-/Rektumkarzinome" als flaechendeckende chirurgische Qualitaetssicherung. Der Chirurg 2002; 73:138-46.  19. Reinacher-Schick A, Pohl M, Schmiegel W. Die medikamentoese Therapie des kolorektalen Karzinoms. Internist (Berl) 2009; 50:1239-52.  20. Roblick UJ, Keller R, Hildebrand P, Czymek R, Bruch HP. Qualitaetsstrukturen und Mindestmengen in der Kolon- und Rektumchirurgie. Der Chirurg 2007; 78:989-93.  21. Schuette J. Unterversorgung in der Onkologie. Der Onkologe 2008; 14:701-6.  22. Thermann P, Seufferlein T. Aktuelle Therapie des Kolonkarzinoms. Der Gastroenterolge 2010; 5:57-71.  23. Wedding U, Hoeffgen K, Friedrich C, Pientka L. Versorgungsforschung und Geriatrie: Defizite und Forschungsansaetze am Beispiel des kolorektalen Karzinoms und der Anaemie. Zeitschrift fuer aerztliche Fortbildung und Qualitaetssicherung im Gesundheitswesen 2007; 101:587-92.  24. Wittekind C. Verschiedene Aspekte des onkologischen Qualitaetsmanagements in der Pathologie. Onkologe 2008; 14 1268-75. | |  |
