# Supplementary material for: Evaluating the Quality of Colorectal Cancer Care across the Interface of Healthcare Sectors
Source: PLoS One. 2013 May 1;8(5):e60947. doi: 10.1371/journal.pone.0060947 (PMC3641026; doi:10.1371/journal.pone.0060947)
Supplement: Table S3 — Systematic literature search – search terms and procedures. (DOCX) [file pone.0060947.s003.docx]

**Table S3: Systematic literature search – search terms and procedures (Ovid Research, MEDLINE; April 6th 2010)**

|  | Searches | Hits |
| --- | --- | --- |
| 1 | diagnosis/ or *"diagnostic techniques and procedures"/ or *diagnostic imaging/ or *tomography, x-ray computed/ or *colonography, computed tomographic/ or *four-dimensional computed tomography/ or *tomography, spiral computed/ or *magnetic resonance imaging/ or *angio-graphy/ or *portography/ or *radiography, abdominal/ or *radiography, thoracic/ or *mass chest x-ray/ or *ultrasonography/ or *endosonography/ or *diagnostic techniques, digestive system/ or *endoscopy, digestive system/ or *endoscopy, gastrointestinal/ or *colonoscopy/ or *sigmoidoscopy/ or *proctoscopy/ or *diagnostic techniques, surgical/ or *biopsy/ or *sentinel lymph node biopsy/ or *laparoscopy/ or *cystoscopy/ or *diagnostic tests, routine/ or *disability evaluation/ or *work capacity evaluation/ or *genetic testing/ or *physical examination/ or *early diagnosis/ or *"early detection of cancer"/ or *"laboratory techniques and procedures"/ or *clinical chemistry tests/ or *blood chemical analysis/ or *prognosis/ or *disease-free survival/ | 347697 |
| 2 | exp Colorectal Surgery/ | 1406 |
| 3 | pathology/ or pathology, clinical/ or pathology, surgical/ | 32921 |
| 4 | Neoplasm Staging/ | 91413 |
| 5 | *Carcinoembryonic Antigen/ | 6350 |
| 6 | microsatelite instability.mp. | 8 |
| 7 | *Neoplasm Staging/ | 4157 |
| 8 | *pathology, clinical/ or *pathology, surgical/ | 3656 |
| 9 | *behavior control/ or *combined modality therapy/ or *chemotherapy, adjuvant/ or *neoadju-vant therapy/ or *radiotherapy, adjuvant/ or *antineoplastic protocols/ or *antineoplastic combined chemotherapy protocols/ or *chemotherapy, cancer, regional perfusion/ or *patient care/ or *aftercare/ or *ambulatory care/ or *palliative care/ or *perioperative care/ or *intraopera-tive care/ or *postoperative care/ or *radiotherapy/ or *rehabilitation/ or *surgical procedures, operative/ or *colectomy/ or *proctocolectomy, restorative/ or *enterostomy/ or *cecostomy/ or *colostomy/ or *hepatectomy/ or *lymph node excision/ or *surgical procedures, elective/ or *surgical procedures, minimally invasive/ | 203291 |
| 10 | polypectomy.mp. | 2627 |
| 11 | *interdisciplinary communication/ or *reminder systems/ or *registries/ or *seer program/ or *documentation/ or *classification/ | 22232 |
| 12 | patient care team/ or *patient-centered care/ | 46691 |
| 13 | *Oncologic Nursing/ | 3561 |
| 14 | *Antibodies, Neoplasm/tu [Therapeutic Use] | 608 |
| 15 | *Neoplasm Metastasis/im, ra, rt, su, th, us [Immunology, Radiography, Radiotherapy, Surgery, Therapy, Ultrasonography] | 999 |
| 16 | *health services administration/ or *"organization and administration"/ or *decision making, organizational/ or *hospital administration/ or *management information systems/ or *models, organizational/ or *organizational objectives/ or *risk adjustment/ or *safety management/ or *patient care management/ or *comprehensive health care/ or *patient care planning/ or *critical pathways/ or *"continuity of patient care"/ or *patient-centered care/ or *"delivery of health care"/ | 98705 |
| 17 | patient perspective.mp. | 459 |
| 18 | *"patient acceptance of health care"/ or *patient compliance/ or *patient participation/ or *patient satisfaction/ or *patient preference/ or *treatment refusal/ | 53000 |
| 19 | pathologic$ staging.mp. | 1181 |
| 20 | patient outcome.mp. | 6072 |
| 21 | colonoscopy.mp. | 17725 |
| 22 | proctoscopy.mp. | 1749 |
| 23 | cancer surveillance.mp. | 862 |
| 24 | colon imaging.mp. | 20 |
| 25 | chemotherapy report*.mp. | 134 |
| 26 | pathology report*.mp. | 2000 |
| 27 | operative report*.mp. | 508 |
| 28 | surgical margin*.mp. | 4403 |
| 29 | surgical therapy.mp. | 11204 |
| 30 | *"emigrants and immigrants"/ or *arabs/ | 2340 |
| 31 | race disparity.mp. | 7 |
| 32 | *"Quality of Life"/ | 35992 |
| 33 | cecal intubation.mp. | 138 |
| 34 | National Cancer Data Base.mp. | 208 |
| 35 | patient survey.mp. | 580 |
| 36 | patient questionnaire*.mp. | 1119 |
| 37 | psycho-oncology.mp. | 188 |
| 38 | *age factors/ or *sex factors/ | 5768 |
| 39 | *Cancer Care Facilities/ | 1637 |
| 40 | 1 or 3 or 4 or 5 or 6 or 7 or 8 or 19 | 465357 |
| 41 | 2 or 9 or 10 or 14 or 15 or 28 or 29 | 223303 |
| 42 | 21 or 22 or 24 or 33 or 40 | 476079 |
| 43 | 11 or 12 or 13 or 16 or 25 or 26 or 27 or 34 or 37 or 39 | 167553 |
| 44 | 17 or 18 or 20 or 23 or 30 or 31 or 32 or 35 or 36 or 38 | 104260 |
| 45 | cancer follow up.mp. | 285 |
| 46 | 44 or 45 | 104523 |
| 47 | *Patient Care Planning/ | 9976 |
| 48 | 43 or 47 | 167553 |
| 49 | *Patient Selection/ | 9800 |
| 50 | 46 or 49 | 113835 |
| 51 | patient care management.mp. [mp=title, original title, abstract, name of substance word, subject heading word, unique identifier] | 1809 |
| 52 | 48 or 51 | 168137 |
| 53 | patient care planning.mp. [mp=title, original title, abstract, name of substance word, subject heading word, unique identifier] | 29824 |
| 54 | risk adjustment.mp. [mp=title, original title, abstract, name of substance word, subject heading word, unique identifier] | 2092 |
| 55 | continuity of patient care.mp. [mp=title, original title, abstract, name of substance word, subject heading word, unique identifier] | 11172 |
| 56 | critical pathway.mp. [mp=title, original title, abstract, name of substance word, subject heading word, unique identifier] | 406 |
| 57 | 52 or 53 or 54 or 55 or 56 | 191833 |
| 58 | patient selection.mp. [mp=title, original title, abstract, name of substance word, subject heading word, unique identifier] | 45418 |
| 59 | 50 or 58 | 148633 |
| 60 | exp Colorectal Neoplasms/ | 117386 |
| 61 | (colorectal cancer or colorectal carcinoma$ or colorectal tumo?r$ or colorectal neoplasm$ or colorectal polyp$ or rectal cancer or rectal carcinoma$ or rectal tumo?r$ or rectal neoplasm$ or colonic cancer or colonic carcinoma$ or colonic tumo?r$ or colonic neoplasm$ or colon polyp$ or colon cancer or colon tumo?r$ or Sigmoid carcinoma$ or sigmoid cancer or sigmoid neoplasm$ or sigmoid tumo?r$ or bowel cancer or bowel carcinoma$ or bowel tumo?r$ or intestine cancer or intestine carcinoma$ or intestine tumo?r$).mp. | 125772 |
| 62 | 60 or 61 | 131173 |
| 63 | *"Quality of Health Care"/ | 21869 |
| 64 | *Medical Audit/ or *Clinical Audit/ | 5731 |
| 65 | *Clinical Competence/ | 22287 |
| 66 | *guideline adherence/ or *"outcome assessment (health care)"/ or *"process assessment (health care)"/ or *program evaluation/ or *benchmarking/ or *quality indicators, health care/ | 33046 |
| 67 | *Management Audit/ | 1131 |
| 68 | *Total Quality Management/ | 6415 |
| 69 | (quality assurance or clinical indicator$ or performance indicator$ or quality indicator$ or clinical utili?ation or guideline adherence or process assessment or healthcare quality or clinical audit or medical audit or clinical competence or program evaluation or benchmarking or delivery of health care or total quality management or management audit or quality measure$ or quality criter$ or quality assessment or performance indicator$ or indicator$ or assessment or scale or valid$ or audit or Safety indicator$ or clinical indicator$ or health indicator$ or league table$).mp. | 1329450 |
| 70 | *"health care quality, access, and evaluation"/ | 0 |
| 71 | 63 or 64 or 65 or 66 or 67 or 68 or 69 or 70 | 134293 |
| 72 | 42 and 62 and 71 | 3666 |
| 73 | 41 and 62 and 71 | 1250 |
| 74 | 57 and 62 and 71 | 318 |
| 75 | 59 and 62 and 71 | 687 |
