# Supplementary material for: Evaluating the Quality of Colorectal Cancer Care across the Interface of Healthcare Sectors
Source: PLoS One. 2013 May 1;8(5):e60947. doi: 10.1371/journal.pone.0060947 (PMC3641026; doi:10.1371/journal.pone.0060947)
Supplement: Table S4 — Systematic literature search – hits. (DOCX) [file pone.0060947.s004.docx]

**Table S4: Systematic literature search – hits (Medline (Ovid): Quality indicators colorectal cancer, April 6th 2010)**

|  | | without limits | including limits*/ doublets removed |
| --- | --- | --- | --- |
| 1 | Diagnostic Techniques and Procedures | 3.666 Hits | 2.986 Hits |
| 2 | Therapy | 1.250 Hits | 1.071 Hits |
| 3 | Delivery of Care | 318 Hits | 277 Hits |
| 4 | Patient Perspective | 687 Hits | 608 Hits |
|  | | 5.921 Hits | 4.942 Hits |

* limits: date: 1998-current
