# Supplementary material for: Evaluating the Quality of Colorectal Cancer Care across the Interface of Healthcare Sectors
Source: PLoS One. 2013 May 1;8(5):e60947. doi: 10.1371/journal.pone.0060947 (PMC3641026; doi:10.1371/journal.pone.0060947)
Supplement: Table S5 — Systematic literature search - inclusion and exclusion criteria. (DOCX) [file pone.0060947.s005.docx]

**Table S5: Systematic literature search - inclusion and exclusion criteria**

| **Inclusion criteria (E)** | | | |
| --- | --- | --- | --- |
| **Criteria** | | | **Example** |
| E1 | Disease according to definition of CRC | | Diagnosed colon or rectal carcinoma or pathohistologically malignant polyps |
| E 2 | Study refers to a defined quality objective (for example implementation or validation of a quality indicator) | | A quality indicator is specified or can easily be extracted (for example guideline recommendations, evaluation of a diagnostic or therapeutic procedure) |
|  | E2.1 | Diagnostic procedures | For example: colonoscopy, computer tomography, ultrasound, CEA, pathohistological examinations (staging) |
|  | E2.2 | Therapeutic procedures | For example: endoscopic polypectomy of malignant polyps, surgery, chemotherapy (adjuvant, neoadjuvant, palliative), radiotherapy, radiochemotherapy (adjuvant, neoadjuvant, palliative), metastatic surgery and therapy, psychooncology |
|  | E2.3 | Management and coordination | For example: cross-sectional delivery of care, planning of therapy, documentation, follow up, surveillance, cooperation |
|  | E2.4 | Patient perspective (patient-relevant outcomes) | For example: patients’ satisfaction, -acceptance, survival, quality of life |
| **Exclusion criteria (A)** | | | |
| A1 | Disease not according to definition of CRC | | For example: Anal carcinoma |
| A2 | Screening in asymptomatic, symptomatic and patients of risk (Familial polyposis; HNCC) | | For example: screening examinations of special population groups |
| A3 | Study does not refer to a defined quality objective | | For example: measured parameters are not named properly |
| A4 | Publications without full text in English or German | | For example: [Spanish] |
| A5 | Publications, which present only in form of an abstract | | No full text available - abstract only |
| A6 | Publications, which present only in form of letters and/or editorials | | Publication type: editorial |
