# Supplementary material for: Evaluating the Quality of Colorectal Cancer Care across the Interface of Healthcare Sectors
Source: PLoS One. 2013 May 1;8(5):e60947. doi: 10.1371/journal.pone.0060947 (PMC3641026; doi:10.1371/journal.pone.0060947)
Supplement: Table S6 — Systematic literature search – included papers. (DOCX) [file pone.0060947.s006.docx]

**Table S6: Systematic literature search – included papers**

| **Included papers (n= 99)** **- alphabetical order** |
| --- |
| 1. Arditi C, Gonvers JJ, Burnand B, Minoli G, Oertli D, Lacaine F, Dubois RW, Vader JP, Schussele F, Peytremann Bridevaux I, Pittet V, Juillerat P et al. Appropriateness of colonoscopy in Europe (EPAGE II). Surveillance after polypectomy and after resection of colorectal cancer. Endoscopy. 2009; 41(3):209-217 2. Aslinia F, Uradomo L, Steele A, Greenwald BD, Raufman JP. Quality assessment of colonoscopic cecal intubation: an analysis of 6 years of continuous practice at a university hospital. American Journal of Gastroenterology. 2006; 101(4):721-731 3. Ayanian JZ, Zaslavsky AM, Guadagnoli E, Fuchs CS, Yost KJ, Creech CM, Cress RD, O'Connor LC, West DW, Wright WE. Patients' perceptions of quality of care for colorectal cancer by race, ethnicity, and language. J Clin Oncol. 2005; 23(27):6576-6586 4. Baumann W, Schmitz S. Qualitätsmonitoring mit Indikatoren. Perspektiven der Qualitätssicherung (nicht nur) in onkologischen Schwerpunktpraxen. Onkologe. 2008; 14:1260-1267 5. Baxter NN, Ricciardi R, Simunovic M, Urbach DR, Virnig BA. An evaluation of the relationship between lymph node number and staging in pT3 colon cancer using population-based data. Diseases of the Colon & Rectum. 2010; 53(1):65-70 6. Bilimoria KY, Bentrem DJ, Merkow RP, Nelson H, Wang E, Ko CY, Soper NJ. Laparoscopic-assisted vs. open colectomy for cancer: comparison of short-term outcomes from 121 hospitals. Journal of Gastrointestinal Surgery. 2008a; 12(11):2001-2009 7. Bilimoria KY, Bentrem DJ, Nelson H, Stryker SJ, Stewart AK, Soper NJ, Russell TR, Ko CY. Use and outcomes of laparoscopic-assisted colectomy for cancer in the United States. Archives of Surgery. 2008b; 143(9):832-839 8. Bilimoria KY, Bentrem DJ, Stewart AK, Talamonti MS, Winchester DP, Russell TR, Ko CY. Lymph node evaluation as a colon cancer quality measure: a national hospital report card. Journal of the National Cancer Institute. 2008c; 100(18):1310-1317 9. Bittner R, Burghardt J, Gross E, Grundmann RT, Hermanek P, Isbert C, Junginger T, Kockerling F, Merkel S, Moslein G, Raab HR, Roder J et al. Qualitätsindikatoren bei Diagnostik und Therapie des Rektumkarzinoms. Zentralbl Chir. 2007; 132(2):85-94 10. Bjugn R, Casati B, Norstein J. Structured electronic template for histopathology reports on colorectal carcinomas: a joint project by the Cancer Registry of Norway and the Norwegian Society for Pathology. Human Pathology. 2008; 39(3):359-367 11. Borowski DW, Kelly SB, Bradburn DM, Wilson RG, Gunn A, Ratcliffe AA, Northern R. Impact of surgeon volume and specialization on short-term outcomes in colorectal cancer surgery. British Journal of Surgery. 2007; 94(7):880-889 12. Burke CA, Church JM. Enhancing the quality of colonoscopy: the importance of bowel purgatives. Gastrointestinal Endoscopy. 2007; 66(3):565-573 13. Burton S, Brown G, Daniels IR, Norman AR, Mason B, Cunningham D, Royal M. MRI directed multidisciplinary team preoperative treatment strategy: the way to eliminate positive circumferential margins? British Journal of Cancer. 2006; 94(3):351-357 14. Chang GJ, Hu CY, Eng C, Skibber JM, Rodriguez Bigas MA. Practical application of a calculator for conditional survival in colon cancer. J Clin Oncol. 2009; 27(35):5938-5943 15. Chapuis PH, Chan C, Lin BP, Armstrong K, Armstrong B, Spigelman AD, O'Connell D, Leong D, Dent OF. Pathology reporting of resected colorectal cancers in New South Wales in 2000. ANZ Journal of Surgery. 2007; 77(11):963-969 [Erratum appears in ANZ J Surg. 2008 Jan-Feb;78(1-2):112] 16. Church J. Adenoma detection rate and the quality of colonoscopy: the sword has two edges. Diseases of the Colon & Rectum. 2008; 51(5):520-523 17. Conroy T, Bleiberg H, Glimelius B. Quality of life in patients with advanced colorectal cancer: what has been learnt? European Journal of Cancer. 2003; 39(3):287-294 18. Conroy T, Uwer L, Deblock M. Health-related quality-of-life assessment in gastrointestinal cancer: are results relevant for clinical practice? Current Opinion in Oncology. 2007; 19(4):401-406 19. Cooper GS, Kou TD, Reynolds HLJ. Receipt of guideline-recommended follow-up in older colorectal cancer survivors: a population-based analysis. Cancer. 2008; 113(8):2029-2037 20. Crispin A, Birkner B, Munte A, Nusko G, Mansmann U. Process quality and incidence of acute complications in a series of more than 230,000 outpatient colonoscopies. Endoscopy. 2009; 41(12):1018-1025 21. Cserni G. Nodal staging of colorectal carcinomas and sentinel nodes. Journal of Clinical Pathology. 2003; 56(5):327-335 22. Daniels IR, Fisher SE, Heald RJ, Moran BJ. Accurate staging, selective preoperative therapy and optimal surgery improves outcome in rectal cancer: a review of the recent evidence. Colorectal Disease. 2007; 9(4):290-301 23. Derwinger K, Carlsson G, Gustavsson B. Stage migration in colorectal cancer related to improved lymph node assessment. European Journal of Surgical Oncology. 2007; 33(7):849-853 24. Desch CE, McNiff KK, Schneider EC, Schrag D, McClure J, Lepisto E, Donaldson MS, Kahn KL, Weeks JC, Ko CY, Stewart AK, Edge SB. American Society of Clinical Oncology/National Comprehensive Cancer Network Quality Measures. J Clin Oncol. 2008; 26(21):3631-3637 25. Determann MM, Kollenbaum V-E, Henne-Bruns D. Der Nutzen des Fragebogens zur Erfassung der LebensQualität EORTC-QLQ-C30 fuer die psychoonkologische Outcomeforschung. Zentralblatt fuer Chirurgie. 2004; 129(1):14-17 26. Dixon E, Armstrong C, Maddern G, Sutherland F, Hemming A, Wei A, Sherman M, Moore M, McKay A, Urbach D, Labrie M, Gordon L et al. Development of quality indicators of care for patients undergoing hepatic resection for metastatic colorectal cancer using a Delphi process. Journal of Surgical Research. 2009; 156(1):32-38 27. Duffy MJ. Carcinoembryonic antigen as a marker for colorectal cancer: is it clinically useful? Clinical Chemistry. 2001; 47(4):624-630 28. Engel J, Kerr J, Eckel R, Gunther B, Heiss M, Heitland W, Jauch KW, Siewert JR, Holzel D. Quality of treatment in routine care in a population sample of rectal cancer patients. Acta Oncol. 2005; 44(1):65-74 29. Fakih MG, Padmanabhan A. CEA monitoring in colorectal cancer. What you should know. Oncology (Williston Park). 2006; 20(Williston Park):579-587 30. Foo W, Young JM, Solomon MJ, Wright CM. Family history? The forgotten question in high-risk colorectal cancer patients. Colorectal Disease. 2009; 11(5):450-455 31. Gagliardi AR, Simunovic M, Langer B, Stern H, Brown AD. Development of quality indicators for colorectal cancer surgery, using a 3-step modified Delphi approach. Canadian Journal of Surgery. 2005; 48(6):441-452.[101] 32. Germer CT, Isbert C. Lebensqualität nach Rektumkarzinomoperationen. Chirurg. 2009; 80(4):316-323 33. Gorard DA, McIntyre AS. Completion rate to caecum as a quality measure of colonoscopy in a district general hospital. Colorectal Disease. 2004; 6(4):243-249 34. Gosens MJ, van K, Marijnen CA, Meershoek Klein K, Putter H, Rutten HJ, Bujko K, van d, Nagtegaal ID, Cooperative C. Improvement of staging by combining tumor and treatment parameters: the value for prognostication in rectal cancer. Clinical Gastroenterology & Hepatology. 2007; 5(8):997-1003 35. Habib MR, Solomon MJ, Young JM, Armstrong BK, O'Connell D, Armstrong K. Evidence-based and clinical outcome scores to facilitate audit and feedback for colorectal cancer care. Dis Colon Rectum. 2009; 52(4):616-622 36. Hassan I, Cima RC, Sloan JA. Assessment of quality of life outcomes in the treatment of advanced colorectal malignancies. Gastroenterology Clinics of North America. 2006; 35(1):53-64 37. Junginger T, Hermanek P, Oberholzer K, Schmidberger H. Rektumkarzinom: Behandeln wir zu häufig neoadjuvant? Vorschläge zu einer selektiveren, MRT-basierten Indikation. Zentralblatt für Chirurgie. 2006; 131(4):275-284 38. Jüttner S, Kohout K, Koch HK. Identifikation von Verbesserungspotenzial in der Diagnostik kolorektaler Karzinome mittels retrospektiver Analyse. Z Gastroenterol. 2006; 44(4):297-304 39. Kopp I, Koller M, Rothmund M, Lorenz W, Mitglieder des Qualitätszirkels. Evaluation der Therapie von Patienten mit Rektumkarzinom. Ziele des Heilens (Outcomes) und Implementierung des Konzepts Lebensqualität in die medizinische Gesamtversorgung. Zentralblatt für Chirurgie. 2000; 125(12):940-946 40. Kube R, Ptok H, Wolff S, Lippert H, Gastinger I. Quality of medical care in colorectal cancer in Germany. Onkologie. 2009; 32(1-2):25-29 41. Lippert H, Gastinger I. Versorgung von Patienten mit Rektumkarzinomen in Deutschland. Deutsches Aerzteblatt. 2006; 103(41):A-2709 42. Loffeld RJ, van der Putten AB. The completion rate of colonoscopy in normal daily practice: factors associated with failure. Digestion. 2009; 80(4):267-270 43. Lupinacci R, Penna C, Nordlinger B. Hepatectomy for resectable colorectal cancer metastases--indicators of prognosis, definition of resectability, techniques and outcomes. Surgical Oncology Clinics of North America. 2007; 16(3):493-506 44. MacDermid E, Hooton G, MacDonald M, McKay G, Grose D, Mohammed N, Porteous C. Improving patient survival with the colorectal cancer multi-disciplinary team. Colorectal Disease. 2009; 11(3):291-295 45. Mack LA, Temple WJ. Education is the key to quality of surgery for rectal cancer. European Journal of Surgical Oncology. 2005; 31(6):636-644 46. Malin JL, Asch SM, Kerr EA, McGlynn EA. Evaluating the quality of cancer care: development of cancer quality indicators for a global quality assessment tool. Cancer. 2000; 88(3):701-707 47. Malin JL, Schneider EC, Epstein AM, Adams J, Emanuel EJ, Kahn KL. Results of the national initiative for cancer care quality: How can we improve the quality of cancer care in the United States? Journal of Clinical Oncology. 2006; 24(4): 626-34 48. Maughan NJ, Morris E, Forman D, Quirke P. The validity of the Royal College of Pathologists' colorectal cancer minimum dataset within a population. British Journal of Cancer. 2007; 97(10):1393-1398 49. McGory ML. Quality indicators for the care of colorectal cancer in vulnerable elders. Journal of the American Geriatrics Society. 2007; 55 50. McGory ML, Shekelle PG, Ko CY. Development of quality indicators for patients undergoing colorectal cancer surgery. Journal of the National Cancer Institute. 2006; 98(22):1623-1633 51. McGrath DR, Leong DC, Gibberd R, Armstrong B, Spigelman AD. Surgeon and hospital volume and the management of colorectal cancer patients in Australia. ANZ Journal of Surgery. 2005; 75(10):901-910 52. McNiff KK, Neuss MN, Jacobson JO, Eisenberg PD, Kadlubek P, Simone JV. Measuring Supportive Care in Medical Oncology Practice: Lessons Learned From the Quality Oncology Practice Initiative. J Clin Oncol. 2008; 26(23):3832-3837 53. MERCURY Study Group. Extramural depth of tumor invasion at thin-section MR in patients with rectal cancer: results of the MERCURY study. Radiology. 2007; 243(1):132-139 54. Merkel S, Mansmann U, Hohenberger W, Hermanek P. Einheitliche Berechnung von Lokalrezidivraten-Voraussetzung für das Qualitätsmanagement beim Rektumkarzinom. Z Aerztl Fortbild Qualitaetssich. 2006; 100(3):183-187 55. Mitchell PJ, Ravi S, Grifftiths B, Reid F, Speake D, Midgley C, Mapstone N. Multicentre review of lymph node harvest in colorectal cancer: are we understaging colorectal cancer patients? International Journal of Colorectal Disease. 2009; 24(8):915-921 56. Morris E, Haward RA, Gilthorpe MS, Craigs C, Forman D. The impact of the Calman-Hine report on the processes and outcomes of care for Yorkshire's colorectal cancer patients. British Journal of Cancer. 2006; 95(8):979-985 57. Myles JL, Shamanski F, Witte D. The physicians quality reporting initiative: measure development, implementation and current procedural terminology coding. Advances in Anatomic Pathology. 2010; 17(1):49-52 58. Nagtegaal ID, van der Velde CJ, van der Worp E, Kapiteijn E, Quirke P, van Krieken JH. Macroscopic evaluation of rectal cancer resection specimen: clinical significance of the pathologist in quality control. J Clin Oncol. 2002; 20(7):1729-1734 59. Neuss MN, Desch CE, McNiff KK, Eisenberg PD, Gesme DH, Jacobson JO, Jahanzeb M, Padberg JJ, Rainey JM, Guo JJ, Simone JV. A process for measuring the quality of cancer care: the Quality Oncology Practice Initiative. J Clin Oncol. 2005; 23(25):6233-6239 60. NHS: National Cancer Action Team. Best practise guideline: Amended Colorectal Cancer Measures. National Health Service 2010 61. Nicholl MB, Wright BE, Conway WC, Aarnes Leong T, Sim MS, Faries MB. Does specialized surgical training increase lymph node yield in colon cancer? American Surgeon. 2009; 75(10):887-891 62. Ouwens M, Hermens R, Hulscher M, Vonk-Okhuijsen S, Tjan-Heijnen V, Termeer R, Marres H, Wollersheim H, Grol R. Development of indicators for patient-centred cancer care. Support Care Cancer. 2009 63. Paquette IM, Kemp JA, Finlayson SR. Patient and hospital factors associated with use of sphincter-sparing surgery for rectal cancer. Diseases of the Colon & Rectum. 2010; 53(2):115-120 64. Park IJ, Choi GS, Lim KH, Kang BM, Jun SH. Multidimensional analysis of the learning curve for laparoscopic resection in rectal cancer. Journal of Gastrointestinal Surgery. 2009; 13(2):275-281 65. Park YA, Lee KY, Kim NK, Baik SH, Sohn SK, Cho CW. Prognostic effect of perioperative change of serum carcinoembryonic antigen level: a useful tool for detection of systemic recurrence in rectal cancer. Annals of Surgical Oncology. 2006; 13(5):645-650 66. Patwardhan MB, Samsa GP, McCrory DC, Fisher DA, Mantyh CR, Morse MA, Prosnitz RG, Cline KE, Gray RN. Cancer care quality measures: diagnosis and treatment of colorectal cancer. Evidence Report/Technology Assessment. 2006; 116(138):1 67. Pawlik TM, Assumpcao L, Vossen JA, Buijs M, Gleisner AL, Schulick RD, Choti MA. Trends in nontherapeutic laparotomy rates in patients undergoing surgical therapy for hepatic colorectal metastases. Annals of Surgical Oncology. 2009; 16(2):371-378 68. Peeters KC, van de Velde CJ. Surgical quality assurance in rectal cancer treatment: the key to improved outcome. European Journal of Surgical Oncology. 2005; 31(6):630-635 69. Penninckx F. Surgeon-related aspects of the treatment and outcome after radical resection for rectal cancer. Acta Gastroenterologica Belgica. 2001; 64(3):258-262 70. Petersson LM, Berglund G, Brodin O, Glimelius B, Sjoden PO. Group rehabilitation for cancer patients: satisfaction and perceived benefits. Patient Education & Counseling. 2000; 40(3):219-229 71. Pheby DF, Levine DF, Pitcher RW, Shepherd NA. Lymph node harvests directly influence the staging of colorectal cancer: evidence from a regional audit. Journal of Clinical Pathology. 2004; 57(1):43-47 72. Prandi M, Lionetto R, Bini A, Francioni G, Accarpio G, Anfossi A, Ballario E, Becchi G, Bonilauri S, Carobbi A, Cavaliere P, Garcea D et al. Prognostic evaluation of stage B colon cancer patients is improved by an adequate lymphadenectomy: results of a secondary analysis of a large scale adjuvant trial. Annals of Surgery. 2002; 235(4):458-463 73. Prosnitz RG, Patwardhan MB, Samsa GP, Mantyh CR, Fisher DA, McCrory DC, Cline KE, Gray RN, Morse MA. Quality measures for the use of adjuvant chemotherapy and radiation therapy in patients with colorectal cancer: a systematic review. Cancer. 2006; 107(10):2352-2360 74. Prystowsky JB, Bordage G, Feinglass JM. Patient outcomes for segmental colon resection according to surgeon's training, certification, and experience. Surgery. 2002; 132(4):663-670 75. Ptok H, Steinert R, Meyer F, Kroll KP, Scheele C, Kockerling F, Gastinger I, Lippert H. Operative Behandlung von Rektumkarzinomen im Vergleich. Onkologische Langzeitergebnisse einer multizentrischen Beobachtungsstudie nach laparoskopisch-assistierter, konvertierter und primär offener Operation. Chirurg. 2006; 77(8):709-717 76. Rex DK, Bond JH, Winawer S, Levin TR, Burt RW, Johnson DA, Kirk LM, Litlin S, Lieberman DA, Waye JD, Church J, Marshall JB et al. Quality in the technical performance of colonoscopy and the continuous quality improvement process for colonoscopy: recommendations of the U.S. Multi-Society Task Force on Colorectal Cancer. American Journal of Gastroenterology. 2002; 97(6):1296-1308 77. Richter D, Lorenz D, Isemer FE, Braun S, Fisseler Eckhoff A. Acetonbehandlung zur Lymphknotenpräparation beim Staging von kolorektalen Resektaten. Pathologe. 2007; 28(4):269-272 78. Rullier A, Laurent C, Capdepont M, Vendrely V, Belleannee G, Bioulac Sage P, Rullier E. Lymph nodes after preoperative chemoradiotherapy for rectal carcinoma: number, status, and impact on survival. American Journal of Surgical Pathology. 2008; 32(1):45-50 79. Rulyak SJ, Lieberman DA, Wagner EH, Mandelson MT. Outcome of follow-up colon examination among a population-based cohort of colorectal cancer patients. Clinical Gastroenterology & Hepatology. 2007; 5(4):470-476. 80. Sahay TB, Gray RE, Fitch M. A qualitative study of patient perspectives on colorectal cancer. Cancer Practice. 2000; 8(1):38-44 81. Sargent DJ, Patiyil S, Yothers G, Haller DG, Gray R, Benedetti J, Buyse M, Labianca R, Seitz JF, O'Callaghan CJ, Francini G, Grothey A et al. End points for colon cancer adjuvant trials: observations and recommendations based on individual patient data from 20,898 patients enrolled onto 18 randomized trials from the ACCENT Group. J Clin Oncol. 2007; 25(29):4569-4574 82. Scheele J, Altendorf Hofmann A. Resection of colorectal liver metastases. Langenbecks Archives of Surgery. 1999; 384(4):313-327 83. Schiedeck T. Chirurgische Therapie des Rektumkarzinoms. Richtig resezieren und rekonstruieren. MMW Fortschritte der Medizin. 2007; 149(23):29-32 84. Schofield JB, Mounter NA, Mallett R, Haboubi NY. The importance of accurate pathological assessment of lymph node involvement in colorectal cancer. Colorectal Disease. 2006; 8(6):460-470 85. Smith JA, King PM, Lane RH, Thompson MR. Evidence of the effect of 'specialization' on the management, surgical outcome and survival from colorectal cancer in Wessex. British Journal of Surgery. 2003; 90(5):583-592 86. Stojadinovic A, Allen PJ, Protic M, Potter JF, Shriver CD, Nelson JM, Peoples GE. Colon sentinel lymph node mapping: practical surgical applications. Journal of the American College of Surgeons. 2005; 201(2):297-313 87. van Krieken J, Nagtegaal ID. Pathological quality assurance in gastro-intestinal cancer. European Journal of Surgical Oncology. 2005; 31(6):675-680 88. Vather R, Sammour T, Kahokehr A, Connolly AB, Hill AG. Lymph node evaluation and long-term survival in Stage II and Stage III colon cancer: a national study. Annals of Surgical Oncology. 2009; 16(3):585-593 89. Washington MK. Colorectal carcinoma: selected issues in pathologic examination and staging and determination of prognostic factors. Archives of Pathology & Laboratory Medicine. 2008; 132(10):1600-1607 90. Wei JT, Miller EA, Woosley JT, Martin CF, Sandler RS. Quality of colon carcinoma pathology reporting: a process of care study. Cancer. 2004; 100(6):1262-1267 91. Wilson TR, Alexander DJ, Kind P. Measurement of health-related quality of life in the early follow-up of colon and rectal cancer. Diseases of the Colon & Rectum. 2006; 49(11):1692-1702 92. Wittekind C, Tischoff I. Kolorektales Karzinom: Histopathologische Fehler – Welche Bedeutung haben sie für die Stadieneinteilung und Therapie? Zentralblatt für Chirurgie. 2006; 131(2):157-161 93. Wong SL, Ji H, Hollenbeck BK, Morris AM, Baser O, Birkmeyer JD. Hospital lymph node examination rates and survival after resection for colon cancer. JAMA. 2007; 298(18):2149-2154 94. Wright FC, Law CH, Berry S, Smith AJ. Clinically important aspects of lymph node assessment in colon cancer. Journal of Surgical Oncology. 2009; 99(4):248-255 95. Wright FC, Law CH, Last L, Khalifa M, Arnaout A, Naseer Z, Klar N, Gallinger S, Smith AJ. Lymph node retrieval and assessment in stage II colorectal cancer: a population-based study. Annals of Surgical Oncology. 2003; 10(8):903-909 96. Wulf J, Kramer K, van Aaken C, Dietzel F, Lucas D, Pfandner K, Schimpke T, Schulze W, Thiel HJ, Ziegler K, Flentje M. Outcome of postoperative treatment for rectal cancer UICC stage II and III in day-to-day clinical practice. Results from a retrospective quality control analysis in six institutions in North Bavaria (Germany). Strahlentherapie und Onkologie. 2004; 180(1):5-14 97. Xiao H, Lichtman SM. Management of colorectal cancer in older patients. Oncology (Williston Park). 2006; 20:741-750 98. Yusoff IF, Hoffman NE, Ee HC. Colonoscopic surveillance after surgery for colorectal cancer. ANZ Journal of Surgery. 2003; 73(1-2):3-7 99. Zuckerman IH, Rapp T, Onukwugha E, Davidoff A, Choti MA, Gardner J, Seal B, Mullins CD. Effect of age on survival benefit of adjuvant chemotherapy in elderly patients with Stage III colon cancer. Journal of the American Geriatrics Society. 2009; 57(8):1403-1410 |
