# Supplementary material for: Evaluating the Quality of Colorectal Cancer Care across the Interface of Healthcare Sectors
Source: PLoS One. 2013 May 1;8(5):e60947. doi: 10.1371/journal.pone.0060947 (PMC3641026; doi:10.1371/journal.pone.0060947)
Supplement: Table S7 — Search of indicator databases. (DOCX) [file pone.0060947.s007.docx]

**Table S7: Search of indicator databases**

| Indicator-systems/-databases | N^o^ of indicators | Date of search |
| --- | --- | --- |
| AAMC - American Association of Medical Colleges (USA) | - | 08.02.2010 |
| ACHS - Australian Council on Healthcare Standards (Australia) | 8 | 08.02.2010 |
| ÄZQ – Ärztliches Zentrum für Qualität in der Medizin (Germany) | - | 08.02.2010 |
| AHCPR – Agency for Health Care Policy and Research (USA) | 25 | 08.02.2010 |
| AHRQ - Agency for Healthcare Research and Quality (USA) | 1 | 08.02.2010 |
| AIHW – Australian Institute of Health and Welfare (Australia) | 13 | 08.02.2010 |
| ANA - American Nurses Association`s Safety and Quality Initiative (USA) | 2 | 08.02.2010 |
| ANAES – Agence nationale d’accréditation et d’évaluation en santé (France) | - | 08.02.2010 |
| AOK-Indikatoren für Arztnetze (Germany) | - | 08.02.2010 |
| AQUIK - Ambulante Qualitätsindikatoren und Kennzahlen (Germany) | - | 08.02.2010 |
| Australian Commission on Safety and Quality in Healthcare (Australia) | 13 | 10.02.2010 |
| Australian Government Department of Health and Ageing (Australia) | 13 | 10.02.2010 |
| BQS - Bundesgeschäftsstelle für Qualitätssicherung (Germany) | - | 10.02.2010 |
| CAHPS - Consumer Assessment of Healthcare Providers and Systems (USA) | 8 | 10.02.2010 |
| CIHI - Canadian Institute for Health Information (Canada) | 1 | 15.02.2010 |
| Care Quality Commission (UK) | 5 | 15.02.2010 |
| CCECQA – Comité de Coordination de l’Evaluation Clinique et de la Qualité en Aquitaine (France) | - | 15.02.2010 |
| Clinical Information Access Programme (Australia) | - | 15.02.2010 |
| CMS - Center for Medicare and Medicaid Services (USA) | 14 | 15.02.2010 |
| Commonwealth Fund (USA) | 8 | 15.02.2010 |
| CRAG – Clinical Resource and Audit Group (Scotland) | 2 | 15.02.2010 |
| Department of Health (UK) | - | 15.02.2010 |
| DK NIP - Det Nationale Indikatorprojekt (Denmark) | - | 15.02.2010 |
| Dr. Foster (UK) | - | 15.02.2010 |
| ESQH - European Society for Quality in Healthcare (International) | 4 | 15.02.2010 |
| Europäische Union (International) | 9 | 15.02.2010 |
| FACCT – Foundation of Accountability (USA) | 11 | 15.02.2010 |
| FOQUAL – Forum de la Qualité (Switzerland) | 1 | 17.02.2010 |
| HCC - Health Care Choices (USA) | 3 | 17.02.2010 |
| Health Canada (Canada) | 9 | 17.02.2010 |
| Health Council of Canada (Canada) | - | 17.02.2010 |
| Helios Klinken (Germany) | 2 | 17.02.2010 |
| HGRD – Health Grades Inc. (USA) | 13 | 17.02.2010 |
| IHI - Institute for Healthcare Improvement (USA) | 3 | 17.02.2010 |
| IQIP – International Quality Indicator Project (International) | 4 | 17.02.2010 |
| JCAHO - Joint Commission on Accreditation of Health Care Organizations Hospital Core Measures (USA) | 2 | 17.02.2010 |
| Kaiser permanente (USA) | 8 | 17.02.2010 |
| Leapfrog – Leapfrog Group for Patient Safety (USA) | - | 18.02.2010 |
| Massachusetts Health Care Quality and Cost Council (USA) | 4 | 18.02.2010 |
| NCQA – National Committee for Quality Assurance (UK) | 11 | 08.02.2010 |
| NHS Clinical and Health Outcomes Knowledge Base (UK) | 6 | 18.02.2010 |
| NHS - National Institute for Health and Clinical Excellence: Guidelines (UK) | - | 18.02.2010 |
| NHS North West (UK) | - | 18.02.2010 |
| NHS Quality and Outcomes Framework (UK) | 12 | 10.02.2010 |
| NICS - National Institute of Clinical Studies (Australia) | - | 18.02.2010 |
| NQMC - National Quality Measures Clearinghouse (USA) | 52 | 12.02.2010 |
| OECD – Organisation of Economic Cooperation and Development (International) | 5 | 17.02.2010 |
| Office of Statewide Healthplanning and Development (USA) | - | 18.02.2010 |
| Ontario Ministry of Health and long- term Care (Australia) | - | 18.02.2010 |
| Premier (USA) | - | 18.02.2010 |
| Public Health Agency of Canada (Canada) | - | 19.02.2010 |
| Quality indicator project - A business unit of the Maryland Hospital Association (USA) | 3 | 19.02.2010 |
| QISA – Qualitätsindikatorensystem für die ambulante Versorgung (Germany) | 2 | 19.02.2010 |
| RAND - Research and Development (USA) | 26 | 22.02.2010 |
| RI - Rhode Island Health Quality Performance Measurement and Reporting Program (USA) | 6 | 22.02.2010 |
| Socialstyrelsen (Sweden) | 3 | 22.02.2010 |
| Statens folkhälsoinstitut (Sweden) | - | 22.02.2010 |
| University of Oxford Patient Reported Outcomes Management (UK) | - | 22.02.2010 |
| US-Department of Health and Human Services (USA) | - | 22.02.2010 |
| Verein Outcome (Switzerland) | - | 23.02.2010 |
| VGHI – Victorian Government Health Information – Clinical indicators in Victoria`s hospitals (Australia) | 2 | 23.02.2010 |
| Word Health Organization | 4 | 23.02.2010 |
| **Sum** | **318** |  |
| Hand search: Indikator-Systems | N^o^ of indicators | Date of search |
| AMA American Medical Association | 41 | 08.02.2010 |
| AQA alliance | 22 | 10.02.2010 |
| NQF National Quality Forum | 4 | 10.02.2010 |
| NHS National Cancer Action Team | 12 | 10.02.2010 |
| CQC Care Quality Commission (UK) | 5 | 12.02.2010 |
| WDC Westdeutsche Darmkrebs Centren | 13 | 23.02.2010 |
| Tumorzentrum Regensburg | 4 | 23.02.2010 |
| **Sum** | **101** |  |
| **Total: 419 indicators** | | |
